# Video in Survey Interviews: Effects on Data Quality and Respondent Experience

**DOI:** 10.12758/mda.2022.13

**Published:** 2023

**Authors:** Frederick G. Conrad, Michael F. Schober, Andrew L. Hupp, Brady T. West, Kallan M. Larsen, Ai Rene Ong, Tianheao Wang

**Affiliations:** 1 Institute for Social Research, University of Michigan, Ann Arbor; 2 Department of Psychology, The New School for Social Research, New York

**Keywords:** live video survey interviews, video mediated interviews, video web surveys, pre-recorded video interviews, survey satisficing, sensitive questions, disclosure of sensitive information

## Abstract

This study investigates the extent to which video technologies – now ubiquitous – might be useful for survey measurement. We compare respondents’ performance and experience (n = 1,067) in live video-mediated interviews, a web survey in which prerecorded interviewers read questions, and a conventional (textual) web survey. Compared to web survey respondents, those interviewed via live video were less likely to select the same response for all statements in a battery (non-differentiation) and reported higher satisfaction with their experience but provided more rounded numerical (presumably less thoughtful) answers and selected answers that were less sensitive (more socially desirable). This suggests the presence of a live interviewer, even if mediated, can keep respondents motivated and conscientious but may introduce time pressure – a likely reason for increased rounding – and social presence – a likely reason for more socially desirable responding. Respondents “interviewed” by a prerecorded interviewer, rounded fewer numerical answers and responded more candidly than did those in the other modes, but engaged in non-differentiation more than did live video respondents, suggesting there are advantages and disadvantages for both video modes. Both live and prerecorded video seem potentially viable for use in production surveys and may be especially valuable when in-person interviews are not feasible.

Since video capability has become standard on computers and smartphones, video communication has become ubiquitous–at least for those with access to the right equipment and connectivity. For many, two-way live video communication has become an indispensable option for remote personal and business communication. One-way video communication has also become commonplace, whether via live streaming (from baby monitors to video doorbells to security surveillance systems) or via the recorded video that has become a fixture of the environment, from television screens in countless public places to online instructional videos to personal videos recorded and posted by smartphone users.

To what extent might video technologies be useful for collecting survey data? Even before video was ubiquitous, survey methodologists investigated the potential of live video for interviewing (Anderson, 2008) and video recordings of interviewers embedded in self-administered questionnaires (e.g., Fuchs, 2009; Fuchs & Funke, 2007; Gerich, 2008; Krysan & Couper, 2003), or both (Jeannis et al., 2013). Since the proliferation of everyday video communication, investigators have compared data quality between traditional modes and live video in the laboratory (Endres et al., 2022) or between traditional modes and embedded recorded video in the field (Haan et al., 2017), concluding that survey data collection using video technologies is feasible and warrants further investigation.

In the current study, we compare two video “interviewing” modes, Live Video and Prerecorded Video (video recordings of an interviewer asking survey questions, embedded in a web survey), with each other and with a conventional web survey, focusing on data quality and respondents’ experience completing the questionnaire. We see these comparisons as particularly important as the COVID-19 pandemic has introduced new health and safety concerns about in-person data collection, compounding in-person interviewing’s continued challenges and increasing interest in alternatives (Schober, 2018)^1^. It is important to better understand how video interviews should be designed and implemented (Schober et al., 2020), how video technologies (live or prerecorded) might affect respondent participation, engagement, disclosure, rapport, or conscientiousness, and how video interviewing (live or recorded) might compare with data collection modes currently in use with respect to access, data quality, or cost.

Our strategy was to compare response quality for the same 36 survey questions in each of these three modes, with questions in the live and prerecorded video modes asked by the same 9 interviewers (a larger number than in prior studies). We examine data quality with four widely used measures of conscientious responding that presumably reflect respondents’ thoughtfulness, i.e., the extent to which respondents are investing full effort in answering rather than taking mental shortcuts or “satisficing” (Krosnick, 1991; Krosnick & Alwin, 1987; C. Roberts et al., 2019; Simon, 1956) and honesty, i.e., providing socially undesirable and likely uncomfortable but also likely truthful answers (e.g., Schaeffer, 2000; Tourangeau & Smith, 1996). To measure thoughtfulness in answering objective factual questions that require numerical responses, we measured the prevalence of rounded (“heaped”) answers, i.e., ending in a zero or a five; in general, unrounded answers are assumed to be more likely to result from deliberate, memory-based thought processes than from estimation (Brown, 1995; Conrad, Brown, & Cashman, 1998), and they have been shown to be more accurate in answers to these kinds of questions (Holbrook et al., 2014).

We measured thoughtfulness in answering multiple questions that use the same response scale, e.g., from “strongly favor” to “strongly oppose,” by looking at the extent to which respondents selected the same option for all statements in a battery (Herzog & Bachman, 1981), on the assumption that at least some differentiation in the answers reflects more thoughtful responding (Krosnick, 1991; Roberts et al., 2019). We measured honest responding^2^ through increased reporting of socially undesirable information such as more visits to pornography sites or more reports of not voting in local elections on the assumption that more embarrassing or stigmatized answers to survey questions are more likely to be true (e.g., Kreuter et al., 2008; Tourangeau & Yan, 2007; Turner et al., 1998). In addition, we use answering a greater proportion of sensitive questions, i.e., fewer refusals to answer them, as additional evidence of honesty.

Our strategy for measuring respondent experience during data collection was to ask post-interview, online debriefing questions about how respondents had felt during the survey and (for the live and prerecorded video respondents) about any technical problems they may have experienced during the interview.

## Features of the Modes and Implications for Response Quality

To develop expectations about how the quality of data collected in the three modes might differ, we have decomposed the modes into (at least some of) their features. This is presented in Table 1. The modes as we implemented them differ on several features, any of which or any combination of which could affect response quality and respondent experience. The values in the table suggest that live video interviews create social presence of the interviewer – a sense that a human interlocutor is present (Lind et al., 2013): respondents and live interviewers can engage in dialogue, and the interviewer’s facial expressions can change based on the respondents’ speech and behavior; the spoken questions and facial movement in prerecorded video may create a weaker sense of social presence. The web survey mode and prerecorded video are self-administered in the sense that the respondent controls the flow of the “interview”; self-administration likely creates a greater sense of privacy for respondents than is present in live video interviews (e.g., Kreuter, Presser & Tourangeau, 2008; Tourangeau & Smith, 1996).

Based on these features, how might live video interviewing affect response quality relative to a web survey? For *thoughtful responding*, the increased social presence of the interviewer in live video could lead respondents to feel more accountable for their answers or, from another perspective, less able to get away with low effort responding, which could lead to less non-differentiation than in a web survey. Endres et al. (2022) observed a similar result in comparing live video interviews to web surveys.

On the other hand, live video interviews could increase *rounding* by creating time pressure and thus quicker responses to avoid awkward silences as in everyday conversation (e.g., Jefferson, 1988; F. Roberts & Francis, 2013). More specifically, increased time pressure may push respondents to replace more time-consuming recall-and-count strategies with faster estimation processes that are more likely to result in rounded answers (Brown, 1995; Conrad et al., 1998; Holbrook et al., 2014).

With respect to *socially desirable responding*, live video could feel more intrusive and create more opportunity for respondents to feel judged than a web survey, potentially leading respondents to produce fewer socially undesirable (i.e., fewer honest) answers and refuse to answer more questions. Endres et al. (2022) also report more disclosure in a web survey than live video interviews.

As for respondent *subjective experience*, the same alternate possibilities are plausible. The increased social presence of the interviewer in live video data collection could lead respondents to be generally more satisfied due to establishing rapport and a sense of connection with interviewers, increasing their willingness to answer honestly (Sun et al., 2020) or it could feel intrusive and less private, reducing satisfaction.

Will prerecorded video feel to respondents more like live video, more like a web survey, or, given that it shares some features with both (Table 1), feel some-where in between? The fact that the prerecorded interviewers speak the survey questions and that their faces are displayed visually and auditorily in the interface, moving as they speak, could activate the same kinds of social responses as might live video interviews, leading to less *non-differentiation* and more honest, i.e., less socially desirable, answers, as well as more positive subjective experiences than in the web survey. But the fact that there is no live interviewer to keep the respondent engaged and accountable or to potentially judge their answers could lead to the same patterns of responding we expect for web surveys^3^. If the latter pattern is observed in our data, it would be consistent with Haan et al.’s (2017) finding of similar levels of socially desirable responding in prerecorded video and web surveys.

## Methods

### Mode Implementations

**Figure 1:**
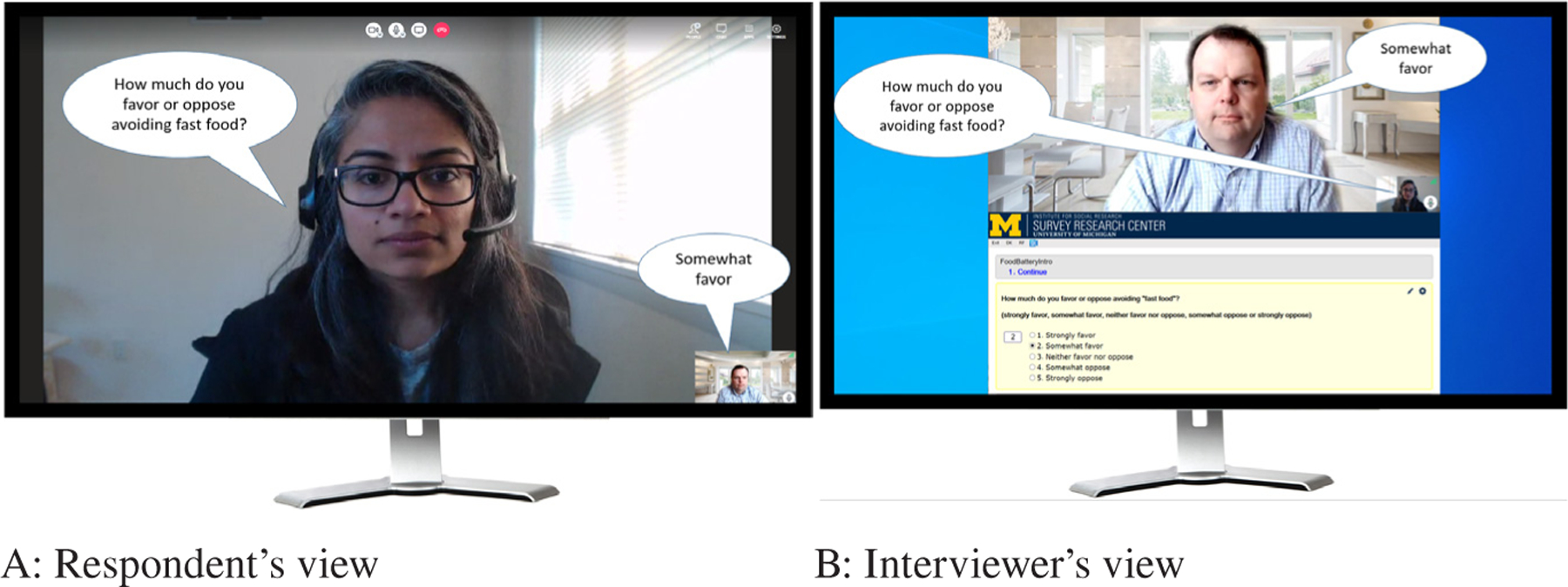
A) Respondent’s screen: Interviewer video fills most of the BlueJeans application window. Respondent’s self-view video thumbnail appears in the lower right corner. Speech bubbles contain text of a question the interviewer asked and a possible answer from the respondent. B) Interviewer’s screen: BlueJeans application window (filled primarily by respondent’s video with interviewer’s self-view video thumbnail in lower right corner) above Blaise instrument. Speech bubbles contain the text of a question that an interviewer asked and a possible answer from the respondent.

All three modes were implemented as a single Blaise 5.6.5 questionnaire which allowed alternate displays appropriate to each mode. Two-way video communication in the Live Video (LV) interviews was conducted via BlueJeans^4^. Except for those on mobile devices, BlueJeans users can join a call through a browser without downloading an app; we expected this to lower the barriers to participation for inexperienced video users. LV respondents were required to schedule the interview beforehand (as opposed to being “cold-called”) using Calendly^5^ software. LV interviews were conducted from a standard call center carrel with a neutral backdrop (see https://www.mivideo.it.umich.edu/media/t/1_1zoid4cu for an example). To give respondents the sense that the interviewer was looking at them while they were answering questions, we positioned the respondents’ video window in the upper half of the interviewer’s screen (above the Blaise questionnaire) so that by looking at the respondent the interviewer was looking in the direction of the camera (see Figure 1). In LV interviews, the interviewer read the question and response options out loud, manually entering answers in the Blaise questionnaire, as an in-person interviewer would do.

Respondents were able to participate on the device of their choice. The percentages of LV respondents who participated on a desktop/laptop computer versus mobile devices appear in Table 5. See Supplementary Appendix A, Figure 1 for screen images of both desktop/laptop and mobile implementations of LV.

The Prerecorded Video (PV) mode was implemented with video recordings^6^ of the same nine interviewers reading the same survey questions embedded in the web display of the Blaise instrument (see https://www.mivideo.it.umich.edu/media/t/1_vjhtigaf for an example). The questions were spoken by the video-recorded interviewers without any textual presentation of the questions. The textually displayed response options appeared automatically on the screen after the video recording of the interviewer reading the question had finished playing. (The on-screen delivery of the response options in PV contrasts with their spoken delivery in LV interviews.)

In the desktop/laptop version, the prerecorded videos autoplayed to reduce the respondent’s effort and to give the delivery of the questions an interviewer-administered character. In the mobile version, this was not possible because autoplay was not implemented in Blaise 5.6 for mobile devices. Thus, these respondents were instructed to click/tap the play button to play each video. Respondents were again able to participate on the device of their choice. The percentages of PV respondents who participated on a desktop/laptop computer versus mobile devices appear in Table 5.

**Figure 2:**
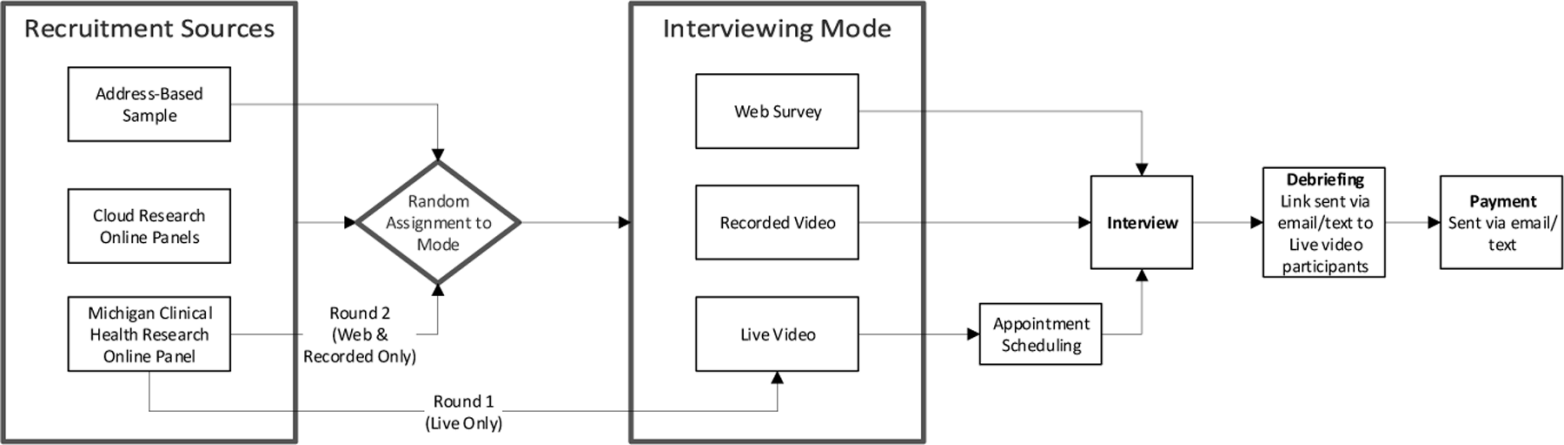
Data collection flow for the full study from recruitment through debriefing and post-paid incentive.

All respondents in PV interviews entered their answers by selecting an option or typing, e.g., an open numerical response. They advanced to the next question by clicking/tapping “Next” (which they could do without answering). See Supplementary Appendix A, Figure 2 for screen images of both desktop/laptop and mobile implementations of PV.

The Web Survey (WS) mode was implemented in Blaise with textually presented questions and response options which appeared on the screen simultaneously (see https://www.mivideo.it.umich.edu/media/t/1_82z2zs7y for an example). The mobile implementation of the WS mode was designed to follow recommended practices for mobile web survey interfaces (Antoun et al., 2018; 2020). In particular, the mobile interface in the WS mode presented large response buttons and large font, fit content to the width of the screen so that horizontal scrolling was not needed, and chose design features that were simple and standard across mobile and desktop operating systems. (In designing the mobile interface for PV interviews, we followed the same design practices to the extent possible, but the screen real estate required us to limit the size of the font and led us to use radio buttons instead of large “clickable” buttons.) Respondents were again able to participate on the device of their choice. The percentages of WS respondents who participated on a desktop/laptop computer versus mobile devices appear in Table 5. See Supplementary Appendix A, Figure 3 for screen images in both desktop/laptop and mobile implementations of WS.

To promote comparability between modes, question batteries were always presented as a series of individual questions even though in the WS mode the batteries could have been implemented as grids. In the PV and WS modes, the display was optimized for screen size, for example using response buttons that included the text of the response within the button for devices with smaller screens, primarily smart-phones, and radio buttons for devices with larger screens, primarily computers.

### Comparing Data Quality Between Modes

We examine data quality in these three modes by measuring the extent to which respondents’ answers were thoughtful, i.e., the extent to which respondents did not take mental shortcuts or “satisfice” (Krosnick, 1991; Krosnick & Alwin, 1987; C. Roberts et al., 2019; Simon, 1956), and the extent to which respondents were willing to disclose sensitive information. We measure thoughtful responding in two ways. First, for questions that require numerical responses we measure the *absence* of thoughtfulness as the prevalence of rounded responses, i.e, non-zero answers that ended in a 0 or a 5 and so were divisible by 5, quantified in two ways: the average percentage of respondents who rounded at least one answer and the average percentage of questions (out of seven) on which rounding is observed.

Second, we measure the absence of thoughtful responding to batteries of questions or statements that use the same response scale, e.g., from “strongly favor” to “strongly oppose,” by classifying instances in which the respondent selected a single response option for all statements in a battery as non-differentiation, and instances in which the respondent selected at least two different responses for different statements in a battery as differentiation; our main dependent variable for measuring data quality was whether a respondent did or did not differentiate between the statements in at least one of the three batteries.

We use greater disclosure of sensitive information (e.g., more reported life-time sexual partners, more reported alcohol use) as evidence of higher quality data, consistent with the evidence that more embarrassing or stigmatized answers are more likely to be true (e.g., Kreuter et al., 2008; Schaeffer, 2000; Tourangeau et al., 2000). We measured disclosure in two ways: the average rated sensitivity of responses to 12 questions concerning potentially sensitive topics and the average number of these questions for which a respondent’s answers were sensitive. We quantified the sensitivity of each response to these 12 questions as the proportion of raters who judged that more than 50% of most people would be very or somewhat uncomfortable selecting that option (See Supplementary Appendix B for details).

### Items

#### Main questionnaire.

Questionnaire items from previously fielded government and social scientific surveys were selected to allow us to test the three main measures of data quality. Supplementary Appendix B lists the 36 items in the questionnaire along with the corresponding data quality indicator (rounding, non-differentiation, disclosure) that each was included to measure. Supplementary Appendix B also details the item selection procedure. Of the 12 items selected to measure disclosure, six were selected because the topics were rated as (1) very or somewhat uncomfortable for most people to be asked by 50% or more of the raters and (2) for which a sensitive response (i.e., which 50% or more of the raters judged would make most people feel very or somewhat uncomfortable) was likely to be selected for a high proportion of respondents based on response distributions from studies that previously used the questions. Six others were selected that concerned topics *not* rated as sensitive but for which a high proportion of respondents was likely to select a sensitive response, based on the same previous studies. The sensitivity of questions increased from the least (for measuring rounding) to most (for measuring disclosure) over the course of the questionnaire. This design was intended to promote completion of the questionnaire and to minimize missing data.

#### Measuring respondent experience.

We quantified respondents’ experience in two ways. First, because the amount of time required to complete a questionnaire has long been used as a measure of respondent burden (e.g., Bradburn, 1979; Hedlin et al., 2005; Office of Management and Budget, 2006; Yan, Fricker, & Tsai, 2020), we calculate mean and median interview duration for the three modes by device type. Second, after respondents completed the main questionnaire, they were directed to an online post-survey questionnaire that included a core set of eight questions about their subjective experience, irrespective of the mode in which they responded to the main questionnaire. This questionnaire included three questions about the interview, two of which were asked only to LV and PV respondents and one of which was asked only to LV respondents, and five questions asked to all respondents about their demographic characteristics. The post-survey questionnaire also included a question about prior use of live video on any device. Most of these items asked respondents to rate their experience on a 5-point scale, with 5 being most positive (see Supplementary Appendix C). Respondents in Live and Prerecorded video interviews were asked if they experienced any of nine technical problems^7^. Another source of data relevant to the experience of LV respondents was transmission logs automatically generated by Bluejeans containing technical information such as video and audio packet loss that might indicate blurred video or choppy audio.

### Interviewers and Interviewer Training

Nine telephone interviewers (median years of interviewing experience = 3.5) conducted the LV interviews during their normal on-site work hours. The same nine interviewers were video-recorded asking the questions; these recordings formed the basis of the PV mode. See Supplementary Appendix D for details about interviewer training, and Schober et al. (2020) for more general considerations about training live video interviewers. The interviewers were all trained in standardized interviewing techniques, designed to reduce interviewer variance by standardizing as much of the data collection as possible.

### Respondent Recruitment

In August 2019 we tested the effectiveness of address-based sampling for all three modes but a low response rate in LV (so low that our budget would not allow recruiting the target number of respondents) led us to shift to opt-in, nonprobability sample sources. One potential downside of recruiting participants from online nonprobability sample sources is that panelists may be more technically proficient than the public in general, but this does not necessarily mean that our participants were any more likely at the time of data collection to have previously participated in live or prerecorded video survey interviews. In addition, it is not possible to fully calculate response rates for samples selected from opt-in, non-probability panels (Callegaro & DiSogra, 2008) because it is generally not known (and was not known to us) how many sample members were exposed to, i.e., read, the invitations sent by the sample vendor. Completion rates – recommended by Callegaro and DiSogra – are presented in Supplementary Appendix E.

The respondents were recruited from two opt-in sample sources, CloudResearch (https://www.cloudresearch.com/) and the Michigan Clinical Health Research (MICHR) (https://michr.umich.edu/), targeting estimated 2018 Current Population Survey (CPS) proportions for cross-classes defined by age, gender, race/ethnicity, and education level, and oversampling adults older than 65 years of age (doubling their proportions) to allow exploratory analyses (not reported here) for this age group. In the end, respondents whose highest level of education was high school or less were underrepresented in all cross-classes for LV; to account for the relatively high level of education in the sample, we adjusted statistically for education level in all mode comparisons. For the PV and WS modes, the CPS targets were reached (see Supplementary Appendix F). Sample members were invited to participate in the three modes at random, with substantially more invitations to participate in a live video interview (see Supplementary Appendix E for the number of invitations and completion rates in each mode for each sample source). We were unable to fulfill our quota for LV respondents from CloudResearch so recruited additional respondents from another opt-in sample source, the Michigan Clinical Health Research (MICHR) panel where we enlisted more LV than PV and WS respondents to compensate for the imbalance in Cloud Research (see Supplementary Appendix G for details about inviting sample members and assigning them to a survey mode). To control for any confounding between sample source and mode we tested the interaction of mode and sample source in all our models; it was never significant, indicating that there was no confound (see Analytic Approach).

Data collection took place between November 2019 and March 2020. See Supplementary Appendix G for further details about recruitment and invitations, incentives, and scheduling constraints.

The total number of completed cases, i.e., cases for which both the main and debriefing questionnaires were submitted, was 1,067. Based on our early experience with Address Based Sampling, we expected sample members assigned to LV interviews to respond at a lower rate than those assigned to the other modes (see Supplementary Appendix E). The number of invitations and the final sample sizes in the three modes for both sample sources appear in Supplementary Appendix E. Note that because we recruited from non-probability, opt-in sample sources, it is not known how many invitations were seen by sample members and thus response rates cannot be calculated, nor can they be interpreted at least comparatively (Callegaro & DiSogra, 2008).

Figure 2 depicts the data collection flow for the full study from recruitment through debriefing and post-paid incentive. Note that LV respondents self-scheduled their interview which necessarily created a lag between screening-in to the study and answering questions; there was no such lag for PV and WS respondents as soon as they had screened in, they were automatically directed to the questionnaire (no scheduling was required because no live interviewers were involved). Thus, it is possible that attrition in LV interviews during the lag could have biased the characteristics of respondents in this mode compared to the other modes. To account for this possibility – and more generally for differences in the characteristics of the responding samples in the three modes – we control for respondent demographics and live video experience in all models (see Analytic Approach).

### Analytic Approach

Our analytic strategy involved fitting models to the variables of interest using GEE (with the xtgee function in Stata/SE 16.0), which allowed us to take interviewer clustering into account in order to compare data quality and respondent experience across modes^8^. For all analyses, we excluded cases (respondents) for which any data relevant to the analysis, e.g., responses to numerical questions for analyses of rounding, were missing.

For each outcome variable of interest, all models included mode as a predictor and all key demographic variables as covariates (respondent age, education, gender, and race), as well as prior respondent experience with live video, sample source (CloudResearch vs. MICHR), device type (desktop/laptop computer vs. smartphone vs. tablet), the two-way interaction of age and mode, and the two-way interaction of sample source and mode. Any variables other than mode, age and sample source that were not significant predictors in the first model were removed in the interest of parsimony, and the models were re-fitted iteratively to include mode, age, sample source, and the remaining significant predictors. Please see Supplementary Appendix H for the terms in all the final models.

The interaction of sample source and mode was included in the initial models to test the possibility that the mode differences were driven by differences between the two sample sources, specifically whether the greater proportion of MICHR than CloudResearch respondents in LV and the greater proportion of CloudResearch than MICHR respondents in PV and the WS modes might have been responsible for the patterns of rounding, non-differentiation, and disclosure. The interaction was not significant in any of the initial models, indicating that mode effects appeared to be robust across the sample sources; in the interest of parsimony, we therefore removed this interaction term from all subsequent models.

The interaction of mode x age was included to control for the possibility that older and younger respondents may have differed in how familiar and comfortable they were with the technology used in the three modes and thus have produced different patterns of data quality across the modes. This interaction was significant and thus included in the final models for all three data quality measures as well as for one battery in which non-differentiation was tested and five of the individual statements in the batteries.

We included the main effect of device in the initial models to control for any differences in data quality that might have originated in the device, such as screen size or input method (e.g., touch versus mouse). The effect was significant for the overall disclosure models and for one of the battery-level models for non-differentiation; therefore the terms were retained in those models.

We measured rounding with two outcome variables. One such measure was an indicator of respondents rounding at least once (1 if rounded on at least one item and 0 if not); each model predicting this outcome treats it as binary and uses a logit link. A second measure was the count of rounded responses for the seven numerical items, which was treated as binomial with seven possible events for each respondent, and a logit model was fitted to these data. The outcome variable measuring non-differentiation is also treated as binary (1 if the respondent selected the same answer for all statements in at least one battery and 0 if the respondent never selected the same answer for all statements in a battery) and modeled using a logit link. One disclosure measure (mean sensitivity of responses to 12 items) followed a normal distribution and so the models treat the measures as numeric; the other disclosure measure (number of responses out of 12 for which the respondent provided a sensitive answer) is treated as binomial and modeled using a logit link.

For items about respondent experience the approach was the same as for data quality. However, technical problems that respondents may have experienced, there were sometimes too few cases for a model to converge. In these situations, we report raw means (i.e., which were not adjusted for covariates) and test comparisons with pairwise t-tests, applying the Bonferroni correction. For the question asked of respondents in only LV, we report raw means.

## Results

### Thoughtful Responding: Rounding

Respondents in LV interviews produced rounded answers, i.e., non-zero answers that ended in a 0 or a 5 and so were divisible by 5, more often than did WS respondents. As shown in the top two rows of Table 2, more respondents rounded at least once and the average number of rounded responses was greater in LV than WS, significantly so for the first measure. And LV respondents produced a (non-significantly) greater percentage of rounded responses than did WS respondents (Row 2).

Where did rounding by PV respondents fall relative to that of LV and WS respondents? By both measures, PV respondents rounded least of all. A significantly lower percentage of PV respondents rounded at least once than did LV respondents, although by these measures PV respondents did not round any more or less than did WS respondents.^9^

The overall pattern is less evident at the level of individual items (rows 3–9) but can be seen, nonetheless. For two of the seven items, a significantly larger percentage of LV respondents rounded their numerical answers than did WS respondents and the pattern was in the same direction for six of the seven items. For two of the seven items, a significantly larger percentage of LV than PV respondents rounded, and the same pattern was evident for six of the seven items.

These mode differences in rounding for individual items are potentially consequential. If the actual survey estimates had been the point of the study (as opposed to mode differences), e.g., mean number of movies watched in the last year, these would have been significantly different in LV than WS for two of the seven items, and different in PV than LV interviews for three of the items (see Supplementary Appendix I).

### Thoughtful Responding: Non-differentiation

Respondents in LV interviews were less likely to select the same answer for all statements in any of the three batteries than were the respondents in the WS and PV modes. As Table 3 details, a significantly smaller proportion of respondents in LV interviews exhibited non-differentiation, even though our implementation of the questionnaire in WS (individual questions for each statement in a battery rather than a grid) may well have reduced non-differentiation among these respondents compared to what might well have resulted with a grid design (e.g., Mavletova et al., 2018). We observed this pattern for all three batteries, significantly so for the money battery;^10^ aggregating the findings for the individual batteries makes the overall pattern (less non-differentiation in LV than in the two self-administered modes) more evident and suggests that LV respondents answered battery items more conscientiously than did respondents in either of the self-administered modes. And, as with rounding, the survey estimates that would have been derived for some statements within each battery – had that been the point of the study – differed significantly by mode, presumably due at least in part to mode differences in non-differentiation (see Supplementary Appendix J). The estimates differed significantly by mode for four of seven statements in the food battery and marginally for a fifth, two of six statements in the money battery, and one of four statements in the sports battery and marginally for two additional statements.

### Honest Responding: Disclosure

By one measure, respondents in LV interviews disclosed significantly less than did WS respondents. As Table 4 shows, across the 12 items selected to measure disclosure, responses in LV were on average less sensitive, i.e., a smaller proportion of judges rated these items as very or somewhat uncomfortable for respondents to select (row 1). By our second measure (row 2), the number of items out of 12 for which the response was rated as very or somewhat uncomfortable by more than 50% of the raters, LV responses were also less sensitive than WS responses, but not significantly so. At the item level, mode differences in the proportion of responses rated very or somewhat uncomfortable to give were significant for four of the twelve items. Disclosure as measured by average response sensitivity for each item appears in Supplementary Appendix K; the pattern of mode differences by this measure closely parallels the pattern for items in Table 4.

PV respondents disclosed significantly more than did respondents in LV when disclosure is measured by mean response sensitivity for the 12 items (row 1) and they disclosed more (but not significantly so) than WS respondents by the same measure. By the other measure (number of items out of 12 for which the response was rated as sensitive) neither mode difference was significant (row 2). For individual items, significantly more PV respondents provided a sensitive answer than did LV respondents for four items and marginally for a fifth.

As with the other data quality measures, the different modes led to significantly different survey estimates (percent of respondents selecting the most sensitive answers) between modes for five items and marginally different estimates for two items (see Supplementary Appendix L). These mode differences in estimates may well be due to how different modes affect disclosure of sensitive information.

### Honest Responding: Item Nonresponse

Levels of item nonresponse (missing answers in cases that completed the debriefing questionnaire) were low overall (0.08% of responses across all modes^11^), but there was significantly more item nonresponse in LV interviews (4.3% of respondents skipped one or more items in this mode) than in the WS (0.5%) mode (two-sided Fisher’s exact test odds ratio 9.01, p < .001). This appears to have been driven by two questions on highly sensitive topics (sex frequency and frequency of visiting a pornography site). The missing data rate for PV interviews (1.8%) was significantly less than the rate for LV interviews (two-sided Fisher’s exact test odds ratio 2.43, p < 0.05) and was marginally greater than for the WS (two-sided Fisher’s exact test odds ratio 3.71, p = 0.08).

## Respondent Experience

### Interview Duration

Our first measure of respondent experience is interview duration, as possible evidence of respondent burden (see Table 5). The WS durations are substantially shorter than the durations for the other two modes (t(957) = 17, p < 0.001) and were particularly brief when respondents participated on their smartphones (t(422)=18, p < 0.001), contrary to prior research indicating longer durations for smartphones (Couper & Peterson, 2017).

### Devices

Device use – which was controlled statistically in the data quality models – varied somewhat by survey mode. See Supplementary Appendix A, Figures 1–3 for screen images of both desktop/laptop and mobile implementations. As shown in Table 5, more respondents participated in LV interviews on a desktop/laptop computer (66.7%) than on a mobile device (31.9% smartphone, 1.4% tablet). It is possible that because LV respondents scheduled an interview for a future day and time and were thus aware of the mode in which they would be interviewed, they chose to participate on a relatively big screen more often than on a mobile device for which screens are generally smaller. PV participants responded on smartphones and tablets somewhat more than LV respondents and WS participants responded on smartphones and computers about equally often. In the two self-administered modes it is unlikely respondents chose their devices based on the interview mode as the screener and interview were continuous in these modes: whatever device these participants used to follow the invitation link was almost certainly the mode in which they were interviewed.

### Satisfaction

After the primary data collection, respondents in all three modes completed an online (self-administered) debriefing questionnaire about their experience participating in the study. LV respondents were significantly more “satisfied with the survey” and a higher proportion were “very satisfied” than were participants in the two self-administered modes (see Table 6), which did not differ from each other. Consistent with this, in response to a question asked only of the LV respondents (results not in the table), 58.5% reported that they “thoroughly enjoyed” their interaction with the interviewer (mean = 4.4 on a 5-point scale). Only two LV respondents (0.7%) reported not enjoying the interview at all. Comparing just the Live and Prerecorded Video modes, LV respondents reported having felt significantly more connected and more comfortable with the interviewer. The higher satisfaction with LV interviews cannot be attributed to greater familiarity with this mode: substantially *fewer* LV respondents (12.3%) reported using live video “weekly or more” than respondents in the WS (27.5%) and PV (24.2%) modes.

### Privacy

More than half of the LV respondents (56.7%) reported that the survey had felt about as private as an in-person interview would have felt in which the interviewer asked the same questions. An additional 26.7% reported that LV felt *more* private than an in-person interview. In contrast, nearly two thirds of the respondents in the self-administered modes reported that the survey had felt more private than an in-person interview; this evidence is consistent with the general assumption that self-administration increases respondents’ sense of privacy (e.g., Tourangeau & Smith, 1996).^12^ Nonetheless, respondents in the three modes did not differ significantly in the extent to which they reported that their answers had been affected by nearby others.

### Technical Problems

More than half of the LV respondents (52.7%) experienced no problems, and of those who experienced any problems, many (45.5%) experienced only one type of problem. As Supplementary Appendix M shows, each of the 11 types of problems was reported rarely, occurring in 2.5% (Volume too soft) to 18.3% (Interrupted speech – interviewer and respondent were speaking at the same time) of interviews; of those reporting any problems, the median number of reported problems was 2.

Follow-up questions about whether and how these technical problems had been resolved (see Supplementary Appendix M) indicated that more problems resolved themselves than with additional intervention by the respondent, interviewer, or others. In whatever way these problems were resolved (or not), the evidence suggests that they were unrelated to respondent satisfaction with the interview; mean respondent satisfaction was not significantly lower (on a 5 point scale) in interviews that had at least one problem (4.52) than in interviews that had none (4.64), *t*(253) = −2.01, p = 0.1. The evidence thus suggests that technical problems were not a major factor in the LV interviews. It is not entirely clear what the technological origins of these problems were, as there was no evidence that the problems in the BlueJeans transmission logs – which were rare – corresponded to respondents’ self-reported technical problems.

## Discussion

These findings demonstrate significant advantages – and disadvantages – for data quality and respondents’ experience in both video modes relative to a conventional web survey, depending on the data quality measure. More specifically, respondents in LV interviews exhibited higher quality data with respect to non-differentiation – they were less likely to select the same answer for all statements in any of the batteries than respondents assigned to the WS mode – but they exhibited lower data quality by rounding more, disclosing less information that was sensitive, and leaving more sensitive questions unanswered. LV respondents reported significantly higher satisfaction with their experience completing the survey than respondents in either of the self-administered modes.

In our view, the overarching explanation for this pattern of findings concerns the presence or absence of a live interviewer. Live interviewers elicited more conscientious responding (less non-differentiation) than was observed in the self-administered modes but seem to have introduced time pressure leading respondents to provide more rounded numerical answers. And the visual and audio presence of a live interviewer who was clearly thinking and reacting in real time very likely led to the lower levels of disclosing sensitive information than in the two self-administered modes. Although a prerecorded video of an interviewer asking questions seemed to evoke a type of social reaction among participants, e.g., feeling comfortable with and connected to the prerecorded interviewer (although less than respondents in LV interviews felt comfortable with and connected to live interviewers), the mode differences seemed largely driven by whether a live human interviewer asked the questions and interacted with the respondent. Data quality and respondent experience did not differ nearly as much between the two self-administered modes.

### Similarity of Live Video Interviewing to In-person Interviewing

Based on the component features of the three modes displayed in Table 1, one would expect the results from LV interviews to be similar to those for in-person interviews (if we had been able to conduct interviews in this mode, despite its greater cost due to interviewer travel expense and the generally higher salaries of field than centralized interviewers). Had we included in-person interviewing in the table, the pattern would have been virtually the same as the pattern for live video interviewing^13^. The primary difference between the features of live video and in-person interviews is that the former mode is mediated and the latter is not, i.e., in in-person interviews, the respondent and interviewer are physically co-present. Yet it is possible that these two modes could differ in how they affect responses and subjective experience. To explore this, we look at published mode comparisons involving in-person interviewing and web surveys, as well as the few studies that compare the results from in-person and live video interviewing.

We observed less non-differentiation in LV interviews than in either the PV or WS modes. This closely mirrors the finding by Heerwegh and Loosveldt (2008) that respondents in in-person interviews exhibited less non-differentiation than those in web surveys, and suggests that the involvement of an interviewer, whether physically or virtually present, motivated respondents to attend to all items in the batteries compared to modes in which respondents self-administer batteries of items.

Similarly, our finding of more rounding in LV interviews than in either the PV or WS modes is analogous to the finding by Liu and Wang (2015) of more rounding when respondents answered feeling thermometer questions in person, i.e., when an interviewer asked the questions, than in web surveys. The authors attributed the greater amount of rounding in in-person interviews to greater time pressure in the former mode than in the web survey – the same mechanism we proposed could lead to more rounding in LV than in the self-administered modes.

The disclosure results underscore how socially present the live video interviewer is despite being mediated; as in in-person interviews, this presence seems to inhibit reporting sensitive information compared to self-administered modes such as CASI and ACASI (e.g., Tourangeau and Smith 1996, and many others) and web surveys (e.g., Burkill et al., 2016; Kreuter et al., 2008). It seems to matter to respondents how they are perceived by the LV interviewer, much as it does in person, even though the video interviewers are not physically co-present. There is to our knowledge one reported comparison of data quality in in-person and live video survey interviews, and it is consistent with our impression that the two modes likely produce data of similar quality: Endres et al. (2022) report no differences between these modes for feeling thermometer items, both of which elicited more socially undesirable (colder) responses that did an online (self-administered) questionnaire. The Endres et al. (2022) finding further supports the conclusion that live video and in-person interviews affect respondents in much the same way and are more similar to each other than to online (self-administered) modes. Certainly, the details of how live video and in-person interviewing affect disclosure across a range of topics should be a top priority in future investigations.

There is evidence that LV respondents’ subjective experience may resemble that of in-person respondents in other studies. Looking first at rapport, the one study that has compared rapport in live video and in-person interviews (Sun et al., 2020) found no difference between the modes in how respondents rated rapport with interviewers. With respect to perceived privacy, our own results indicate that 56.7% of respondents who had participated in a LV interview rated their experienced privacy as being “the same” as in a hypothetical in-person interview.

Finally, it is possible that much as interviewers in in-person interviews are known to introduce error variance, i.e., to create interviewer effects (e.g., Davis, et al., 2010; West & Blom, 2017), the LV interviewers in the current study may have introduced interviewer effects. While we cannot compare the IICs from the current study to those from in-person interviews, it does not appear that the LV interviewers introduced more error variance than is typically observed in in-person interviews: West et al. (2022) analyzed the data collected by interviewers in LV – as well as in PV – and report that interviewer variance (IICs) was low overall, with all IICs less than 0.02.

### Similarity of the two Self-administered Modes

The two self-administered modes are similar to each other in many ways, but as is evident in Table 1, they also differ on several features, primarily those having to do with the presence of an interviewer’s facial and vocal attributes in the PV mode. There is a suggestion in the data that the presence of an interviewer, albeit clearly recorded and asynchronous, may help improve data quality by some measures: while there was less rounding in the two self-administered modes than in LV interviews, rounding was reduced even further in the PV than WS data (the former group of respondents rounded on fewer items than the latter group). It is possible that a video-recorded interviewer may amplify respondents’ willingness to engage in the generally more effortful recall and count process (the likely origin of reduced rounding) than when the interface is entirely textual (i.e., no facial or vocal representation of an interviewer). Similarly, the greater levels of disclosure for several items (Bus Seat, Volunteer, Help Homeless) in PV interviews than in the WS data may also reflect the interviewers’ presence despite their inanimacy. The idea that respondents might react socially to a video recorded interviewer is consistent with Reeves and Nass’s (1996) Computers are Social Actors framework. It is possible that such social engagement might be strengthened and thus disclosure further increased as the feel of a live, two-way interview is approximated. For example, it may be possible to enable respondents in prerecorded video interviews to speak their answers rather than just entering them by clicking and typing (Höhne, 2021). The challenge will be to stop short of reintroducing human-like attributes to the extent that they promote socially desirable responses.

While the data collected in the LV and PV modes were high quality by some measures, the WS mode never produced the highest quality data. In fact, the only measure in which the WS respondents outperformed those in the other two modes is the brevity of data collection sessions. This could be due to inherent properties of the modes, e.g., reading questions may take less time than does the delivery of spoken questions, or to our implementation, in particular allowing respondents in PV to enter their answers only after the video had finished playing. Whatever the origin of the shorter WS sessions, this did not lead to higher satisfaction with the experience, as one would expect if duration were a key determinant of respondent burden (e.g., Bradburn, 1979). Instead, the LV respondents reported greater satisfaction than in the other modes despite significantly longer interview sessions.

### Considerations in Fielding Live Video Interviews

It is possible the preference for LV interviewing is due to the relative novelty of live video communication in general, at least at the time these data were collected when a significantly smaller percentage of respondents in the LV mode reported frequently using live video (weekly or more often) than in the two self-administered modes (see Pew Research Center, 2021). If this is the case, then the preference for live video data collection could fade as the mode becomes widely used in everyday communication. Alternatively, some respondents may just prefer interacting with a live, albeit mediated, interviewer to self-administering survey questions. Yet, for at least some LV respondents the experience was subtly different than in-person interviews: about a quarter reported that they experienced their interview to be more private than a hypothetical in-person interview, consistent with the suggestion that video mediation can provide a “protective barrier,” as observed in training psychologists (Miller & Gibson, 2004). This could bode well for disclosure of sensitive information in live video interviews.

Although LV respondents’ interview experience was quite positive, recruiting sample members to participate in this mode was challenging, particularly from one of the online sample vendors. One consequence of this challenge was that a higher proportion of participants in LV interviews were recruited from the medical research panel than in the two self-administered modes. Might this have accounted for any of our findings? We examined this by testing the interaction of mode and sample source in the initial models developed for all our analyses. This interaction was not significant in any of the models, indicating that the effects of mode were unrelated to the panel from which participants were recruited, supporting the interpretation that the results were in fact due to mode differences.

The combination of greater difficulty recruiting LV respondents and a more positive experience for those who ultimately completed the study in this mode suggests that live video interviews may not be for everyone but are quite appealing to some. It could be that as of now live video interviews fit better into a mixed mode, longitudinal research design, or ongoing panel, where sample members are familiar with and presumably trust the research organization than a stand-alone, cross-sectional study. For example, researchers might initially collect data in a mode with which sample members are familiar, e.g., online, on the telephone, or in an in-person interview, after which researchers would invite sample members to participate in future data collection in a mode of their choice (Conrad et al., 2017) where the choices include live video interviews.

Both live and prerecorded video might be combined in a multimodal data collection platform that takes advantage of the strengths of each mode. For example, researchers might extend the ACASI approach to video interviewing by administering non-sensitive questions in a live video interview in which interviewer and respondent are visible and audible to one another when the questions are not sensitive, but when they are sensitive the questions could be administered in a prerecorded video interview.

Before such hybrid approaches can be developed and deployed with confidence, many questions remain about using video – live or prerecorded – in survey data collection. Will the patterns of findings observed here replicate in other samples, with other recruitment methods, with different survey questions and measures of data quality? Will they replicate with different implementations of these modes? Will the cost saving of live video interviews due to the elimination of travel expenses for in-person interviews be sufficient to offset the additional effort – especially in recruitment – that this mode might entail? Will sample members’ willingness to participate in live video interviews increase as their comfort with live video communication increases (Schober et al., in press), their access to necessary hardware and software increases, and their familiarity with self-scheduling appointments – not just survey interviews – increases? Are there groups of people who might be more likely to participate in a live video interview than in other modes, e.g., those unwilling to invite an interviewer into their home or who live in areas not easily accessible for in-person interviewers? Whatever the answers to these questions, our findings demonstrate that both live and prerecorded video – at least as we implemented them – are viable survey modes with advantages and disadvantages, worth considering as video communication becomes ever more available – and for many people – central to daily life.

## Supplementary Material

appendix

## Figures and Tables

**Table 1:** Features of the three modes that could plausibly affect response quality and respondent experience. Live video interviews and web surveys differ on all these features; prerecorded video shares some features with live video and some with web surveys.

Feature	Live Video	Prerecorded Video	Web Survey
Interview speaks question	Yes	Yes	NA^*^
Respondent speaks answer	Yes	Yes	No
Dialogue between interviewer and respondent	Yes	Yes	No
Facial representation of interviewer	Yes	Yes	Yes
Interviewer’s facial expressions are responsive	Yes	Yes	NA
Questions are self-administered	Yes	Yes	Yes
Questions persist beyond respondent’s first exposure to them	Yes	Yes	Yes
Question is re-presented when…	Interviewer re-reads (aloud) after respondent’s request	Respondent re-plays video as needed	Respondent re-reads as needed
Interviewer has perceptual capability (can see and hear respondent)	Yes	No	NA
Interviewer has evaluative capability (can pass judgment on respondent’s answers)	Yes	No	NA

* NA = Not applicable

**Table 2:** Rounding overall (average percentage of rounded responses and percentage of respondents rounding at least once) and for each of the seven items. Standard errors are in parentheses and p-values less than .05 are **bold**.

		Live Video (n = 278)	Web Survey (n = 403)	Prerecorded Video (n = 385)	Live Video vs. Web Survey	Live Video vs. Prerecorded Video	Prerecorded Video vs. Web Survey
				
					p-value		
*Overall Mode Differences*							
	% Rs rounding at least once	86.9% (2.5%)	82.0% (1.9%)	76.3% (2.2%)	**0.005**	**0.003**	0.773
	Average % rounded responses	28.6% (2.9%)	24.5% (2.2%)	20.9% (2.2%)	0.286	**0.031**	0.209
*Item-Level Mode Differences*							
Television Hours	% Rs who rounded	13.4% (2.0%)	17.9% (1.8%)	17.2% (1.0%)	0.121	0.119	0.726
MovieTheaterYear	% Rs who rounded	10.6% (1.7%)	10.5% (1.6%)	11.0% (1.2%)	0.284	0.472	0.633
MoviesYear	% Rs who rounded	61.0% (3.5%)	54.8% (2.5%)	25.9% (2.5%)	0.175	**<0.001**	**<0.001**
Restaurants Month	% Rs who rounded	30.5% (2.6%)	18.4% (2.0%)	16.8% (1.3%)	**<0.001**	**<0.001**	
SpicyFood	% Rs who rounded	24.3% (2.1%)	21.4% (2.1%)	21.8% (1.4%)	0.366	0.340	0.888
GroceryStore	% Rs who rounded	31.7% (2.8%)	22.0% (2.1%)	26.3% (1.9%)	**0.009**	0.126	0.130
Drinking Water	% Rs who rounded	28.1% (3.1%)	26.8% (2.3%)	27.7% (2.5%)	0.752	0.923	0.797

**Table 3:** Non-differentiation (selecting the same option for all statements in a battery) and mode comparisons overall and for each of the three batteries. Standard errors are in parentheses, and p-values less than .05 are **bold**.

	Live Video (n = 278)	Web Survey (n = 403)	Prerecorded Video (n = 385)	Live Video vs. Web Survey	Live Video vs. Prerecorded Video	Prerecorded Video vs. Web Survey
				
				p-value		
Mode differences overall						
% Rs who selected same option for all questions/statements in at least one battery	1.7% (1.1%)	7.7% (1.3%)	6.0% (1.3%)	**0.018**	**0.027**	0.634
Food Battery (Q8-Q14)						
% Rs who selected same option for all questions	0.5% (0.5%)	1.0% (0.5%)	1.4% (0.6%)	0.470	0.329	0.697
Money Battery (Q15-Q20)						
% Rs who selected same option for all statements	1.1% (0.7%)	4.7% (1.0%)	2.6% (0.7%)	**0.040**	0.254	0.079
Sports Battery (Q26-Q29)						
% Rs who selected same option for all statements	1.1% (0.8%)	3.7% (0.9%)	3.1% (0.9%)	0.110	0.187	0.608

**Table 4:** Mode differences in disclosure overall and for each of 12 sensitive items. Comparisons of the mean sensitivity (percent of raters judging the response as very or somewhat uncomfortable for most people to give) for all 12 responses (row 1) and number of responses out of 12 independently rated as sensitive (very or somewhat uncomfortable for most people to give) by > 50% of online raters (row 2). The values for individual items are the proportion of responses in the current study that were independently rated as sensitive (very or somewhat uncomfortable to give) by > 50% of online raters. Standard errors appear in parentheses, and p-values less than .05 are **bold**.

		Live Video (n = 271)	Web Survey (n = 396)	Prerecorded Video (n = 377)	Live Video vs. Web Survey	Live Video vs. Prerecorded Video	Prerecorded Video vs. Web Survey
				
					p-value		
*Mode differences overall*							
	Mean sensitivity of all 12 responses	0.564 (0.004)	0.581 (0.003)	0.588 (0.003)	**0.001**	**<0.001**	0.110
	Mean number of questions out of 12 for which response is sensitive	3.16 (0.034)	3.20 (0.023)	3.53 (0.029)	0.936	0.498	0.435
*Mode differences for each item*							
Credit Card Balance	Proportion of sensitive responses	37.0% (2.9%)	40.5% (2.5%)	34.3% (1.9%)	0.097	0.381	0.302
Religious Attendance	Proportion of sensitive responses	66.9% (3.6%)	70.2% (2.3%)	74.8% (2.6%)	0.444	0.078	0.180
Bus Seat	Proportion of sensitive responses	36.4% (3.9%)	33.0% (2.3%)	41.8% (3.3%)	0.469	0.299	**0.025**
Volunteer Work	Proportion of sensitive responses	36.5% (3.7%)	46.3% (2.4%)	55.3% (3.0%)	**0.036**	**<0.001**	**0.019**
Help Homeless	Proportion of sensitive responses	38.0% (3.9%)	45.3% (2.5%)	54.3% (3.4%)	0.140	**0.003**	**0.032**
Local Elections	Proportion of sensitive responses	20.2% (2.4%)	28.7% (2.3%)	26.0% (2.0%)	**0.019**	0.086	0.352
Sex Partners Year	Proportion of sensitive responses	49.3% (3.0%)	44.5% (2.5%)	48.4% (2.2%)	0.243	0.814	0.229
Female Sex Partner	Proportion of sensitive responses	100.0%	100.0%	100.0%			
Male Sex Partner	Proportion of sensitive responses	100.0%	100.0%	100.0%			
Sex Frequency	Proportion of sensitive responses	100.0%	100.0%	100.0%			
Sex Partner Gender	Proportion of sensitive responses	74.0% (2.4%)	69.7% (1.5%)	73.6% (1.8%)	0.160	0.891	0.090
Porn Frequency	Proportion of sensitive responses	28.6% (2.2%)	38.0% (2.2%)	38.3% (1.6%)	**0.005**	**0.001**	0.901

**Table 5:** Mean Duration (Mins) and Number of Interviews* by Mode and Device

Device		Live Video	Web Survey	Prerecorded Video	Overall
Computer	Avg. Duration	9.84	7.80	12.43	10.10
	Median	9.38	6.69	10.81	9.08
	# Iws % Within Mode	186 (66.7%)	187 (46.5%)	206 (53.5%)	579 (54.3%)
Smartphone	Avg. Duration	9.93	5.75	13.79	9.48
	Median	9.47	4.97	11.88	8.83
	# Iws % Within Mode	89 (31.9%)	190 (47.3%)	155 (40.3%)	434 (40.7%)
Tablet	Avg. Duration	9.55	6.87	13.42	10.04
	Median	9.73	7.01	10.83	8.96
	# Iws % Within Mode	4 (1.4%)	25 (6.2%)	24 (6.2%)	53 (5.0%)
Total # Iws		279	402	385	1066
Avg. Duration		9.87	6.77	13.04	9.85
Median Duration		9.46	5.85	11.2	9.00

* One case (in Web survey, Computer) was excluded as an outlier; its duration was four times that of the next highest case.

**Table 6:** Mode differences in respondent experience of the interview, measured in an online post-interview debriefing survey (questions are presented in the order they appeared to respondents). Rows marked with # are items for which there were too few cases for the models to converge, and so raw means and test results from pairwise t-tests and Bonferroni correction are reported.

		Live Video (n = 278)	Web Survey (n = 403)	Prerecorded Video (n = 385)	Live Video vs. Web Survey	Live Video vs. Prerecorded Video	Prerecorded Video vs Web Survey
				
					p-value		
Overall, how satisfied were you with this survey?	Mean rating	4.66 (0.096)	4.27 (0.077)	4.20 (0.069)	**<0.001**	**<0.001**	0.332
	Percent Rs “very satisfied”	73.9% (28.1%)	52.1% (19.7%)	52.5% (19.2%)	**<0.001**	**<0.001**	0.904
Overall, how comfortable were you with the interviewer?	Mean rating	4.70 (0.108)	-	4.42 (0.098)	-	**0.004**	-
	Percent Rs “very comfortable”	80.9% (23.7%)	-	69.2% (19.8%)	-	**0.018**	-
How personally connected did you feel to the interviewer?	Mean rating	4.59 (0.17)5	-	3.96 (0.150)		**<0.001**	
	Percent Rs “connected” (4 or 5 on 5-point scale)	88.6% (34.0%)	-	71.4% (18.9%)		**<0.001**	
How often did you feel that you were able to answer the questions honestly?	Mean frequency	1.132 (0.048)	1.158 (0.024)	1.138 (0.018)	0.582	0.885	0.470
	Percent “always”	89.5% (20.5%)	87.1% (15.1%)	89.4% (12.0%)	0.296	0.948	0.244
Imagine you had been asked the survey questions in person, that is, in a face-to-face interview. Did the survey you just completed feel more private, the same, or less private than being asked the questions face-to-face?	Percent “more private”	23.4% (15.8%)	58.3% (10.6%)	56.7% (10.4%)	**<0.001**	**<0.001**	0.655
	Percent “same”	75.0% (15.3%)	45.1% (13.3%)	45.5% (14.4%)	**<0.001**	**<0.001**	0.898
Did anyone nearby affect the way you answered the questions?	Percent “Yes, the people nearby affected my answers”#	2.5% (0.9%)	3.0% (0.8%)	1.3% (0.6%)	1.000	0.890	0.338
	Percent “No, no one was around”	59.6% (24.2%)	51.0% (21.1%)	57.1% (20.1%)	**0.038**	0.522	0.083
How sensitive did you feel the survey questions were?	Percent R’s “very sensitive”	16.5% (26.6%*)	19.5% (19.9%*)	21.8% (16.5%)	0.469	0.128	0.540
How often do you participate in live video calls on any device?	Percent Rs weekly or more	11.7% (25.8%*)	26.7% (13.9%)	23.2% (10.0%)	**<0.001**	**<0.001**	0.148
Were you doing something else during the interview?	Percent Rs “yes”#	4.7% (1.3%)	7.7% (1.3%)	4.7% (1.1%)	0.295	1.000	0.212

## Footnotes

1. We use “in-person” for interviews with physically copresent participants rather than “face-to-face,” as live video certainly involves faces, potentially amplifying their importance compared to in-person interactions.

2. We use the term “honest” though we recognize that more socially desirable responding can occur for many reasons and does not necessarily involve a conscious intention to mislead (e.g., Schaeffer, 2000; Schober & Glick, 2011).

3. For a full list for each measure of how patterns of responses in PV could correspond to the patterns in LV and WS responding in this study, see our Open Science Foundation pre-registration, https://osf.io/2vmx4/?view_only=c90cd24fb46a42d38b285f3453483a37

4. Versions 2.15 to 2.18. We restricted the study to one platform in order to reduce operational complexity, aware that this might reduce participation among users unfamiliar with the platform (see Schober et al., 2020).

5. Calendly is continuously updated, so is not identified by version number.

6. Recorded and edited using Camtasia version 2019.0.5.4959

7. We note that technical problems can occur for many reasons that are not under the researchers’ control, including the respondent’s device and its current level of performance, the respondent’s connection speed, network stability and performance, and presumably internet and platform traffic. These can all be affected by the respondent’s circumstances at the moment of the interview, for instance the number of simultaneous users on the respondent’s network and the resource demands of the simultaneous tasks, ambient noise in the respondent’s environment, and even the respondent’s ability to troubleshoot technical problems on their own.

8. While it is common to model interviewer effects using multilevel models that include random effects of the interviewers, our interest here was in accounting for possible clustering of responses by interviewers in the marginal comparisons between the three modes, not in estimating interviewer variance components. See West et al. (2022) for estimates of interviewer variance components in the data set on which the current article is based.

9. The pattern of results is essentially the same if we define rounding as answers divisible by 10, rather than by 5. The mode comparison p-values are lower (now significant for PV versus WS responses) when rounding is defined as divisible by 10.

10. The pattern of results is essentially the same if we relax the criterion for what counts as non-differentiation so that providing the same response for all or all but one statement in a battery is counted, although the effects are attenuated. LV interviews led to significantly less of this liberally defined non-differentiation than did the WS mode, and less (though not significantly less) of this behavior than in PV interviews.

11. Responses for 30 out of 39,479 possible responses were missing.

12. A small percentage of respondents in all three modes (69 of 1067) reported that this survey had felt less private than an in-person interview (10.4% in LV, 6.0% in PV, 4.2% in the web survey). We can only speculate about why these respondents might see any of these modes as less private than an in-person interview, but perhaps the fact that they are technology-mediated raises the possibility for respondents that their answers may not be secure or that the data collection itself might be subject to surveillance.

13. See Schober et al., in press, for such an analysis of in-person interviews.
