# Supplementary material for: Video in Survey Interviews: Effects on Data Quality and Respondent Experience
Source: Methoden Daten Anal. Author manuscript; Available in PMC 2023 Sep 18. (PMC10506842; doi:10.12758/mda.2022.13)
Supplement: appendix [file NIHMS1846806-supplement-appendix.pdf]

methods, data, analyses | 2022, pp. 1-23 DOI: 10.12758/mda.2022.13

Online-Appendix

Video in Survey Interviews: Effects on Data Quality and Respondent Experience

Supplementary Appendix A
Respondent's view by device type 2

Supplementary Appendix B
Item selection and questionnaire 4

Supplementary Appendix C
Online debriefing items administered immediately after respondents completed the survey 10

Supplementary Appendix D
Interviewer training details 12

Supplementary Appendix E
Invitations and completions by sample source 13

Supplementary Appendix F
Sample Composition by Gender, Age, Race and Education 14

Supplementary Appendix G
Recruitment Procedure, Incentives and Scheduling 15

Supplementary Appendix H
Terms Used in Final Models 16

Supplementary Appendix I
Numerical Estimates by Mode 18

Supplementary Appendix J
Percent "Strongly Favor" or "Strongly Agree" by mode; differences presumably due at least in part to mode differences in non-differentiation 19

Supplementary Appendix K
Mean sensitivity of the selected responses for each item, where sensitivity is measured by the percent of online raters judging each response as very or somewhat uncomfortable for most people to give 20

Supplementary Appendix L
Prevalence Estimates for Sensitive Behaviors by Mode 21

Supplementary Appendix M
Technical Problems 22

References 23

© The Author(s) 2022. This is an Open Access article distributed under the terms of the Creative Commons Attribution 3.0 License. Any further distribution of this work must maintain attribution to the author(s) and the title of the work, journal citation and DOI.

Supplementary Appendix A

Respondent's view by device type

1) Live Video

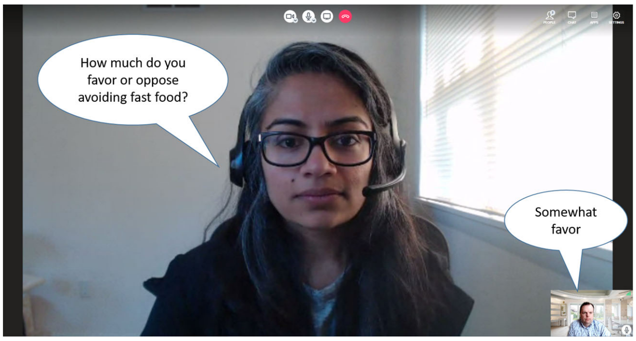

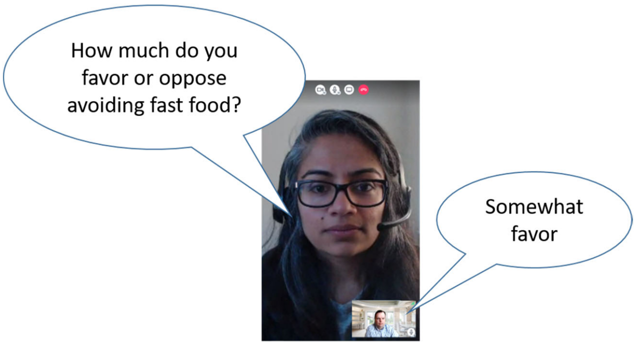

A) Respondent's desktop screen

B) Respondent's mobile screen

Interviewer video fills most of the respondent's display. Respondent's self-view video thumbnail appears in the lower right corner. Speech bubbles contain text of a question the interviewer asked and a possible answer from the respondent.

2) Prerecorded Video

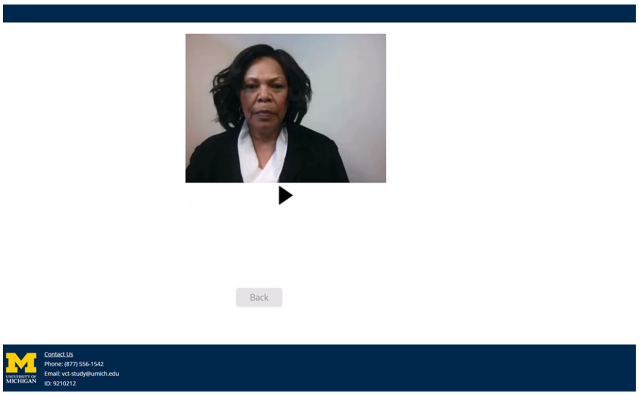

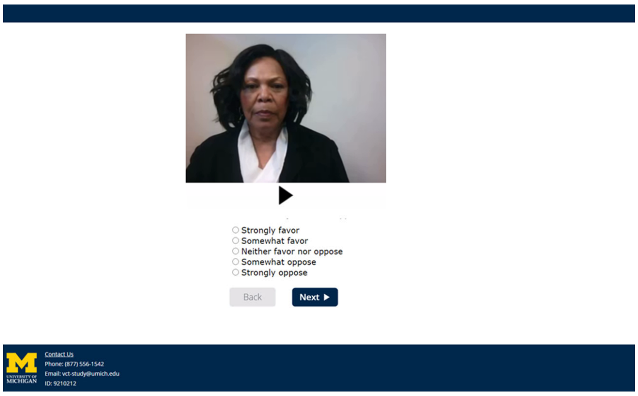

A) Respondent's desktop screen before the video of the interviewer reading the question is played (response options are hidden)

B) Respondent's desktop screen after the video of the interviewer reading the question is played (response options are displayed)

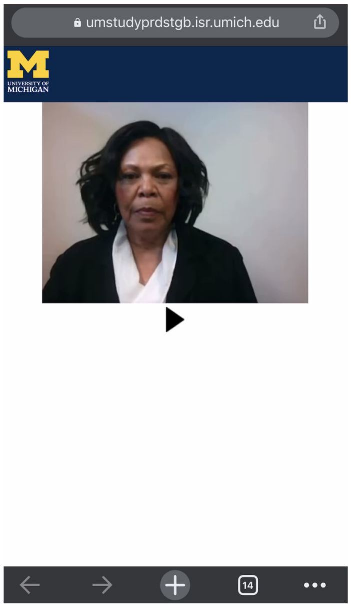

C) Respondent's mobile screen before the video of the interviewer reading the question is played (response options are hidden)

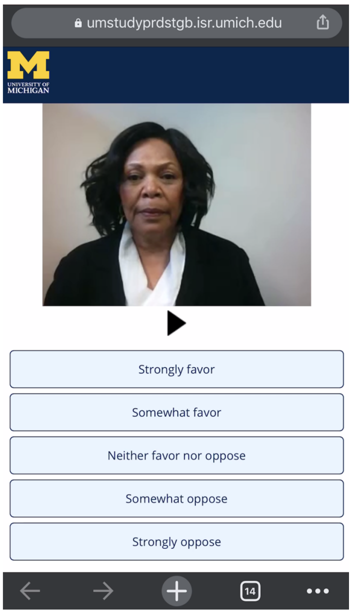

D) Respondent's mobile screen after the video of the interviewer reading the question is played (response options are displayed)

3) Web Survey

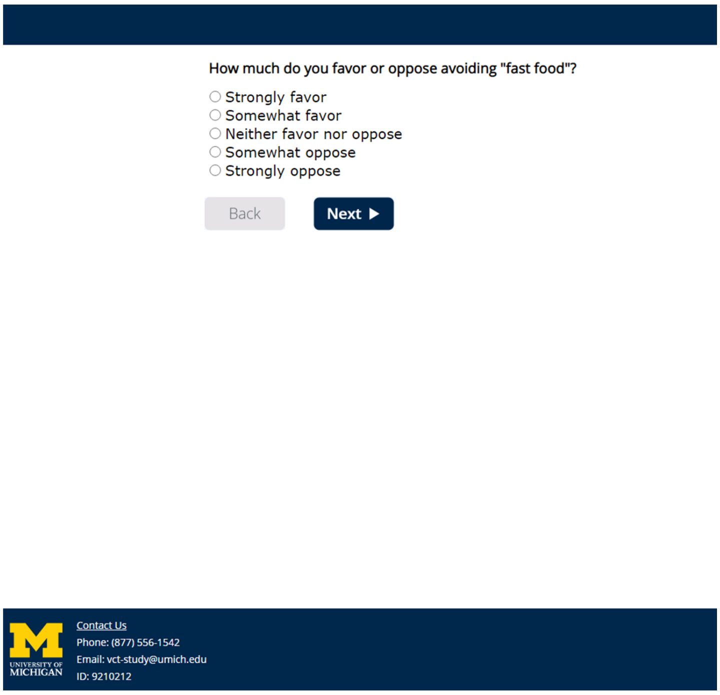

A) Respondent's desktop screen

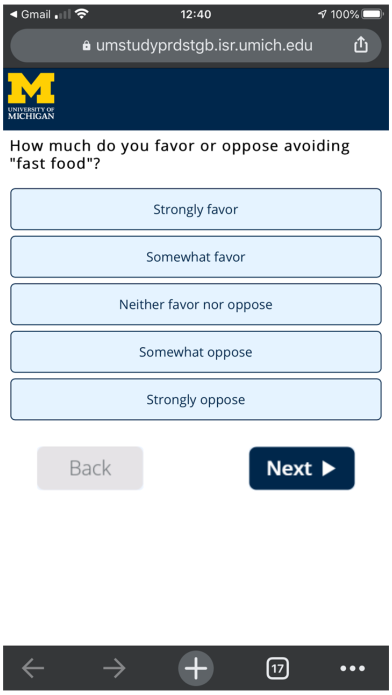

B) Respondent's mobile screen

Supplementary Appendix B

Item selection and questionnaire

Items relevant to current hypotheses from the Schober et al. (2015) and Lind et al. (2013) mode comparison studies, which themselves had been drawn from previously-fielded government and social scientific surveys, were considered, along with additional potential items listed in the Q-Bank repository (https://wwwn.cdc.gov/QBANK/Home.aspx) that would allow for the measurement of rounding and disclosure (e.g., questions that started with "How many"). In order to further test non-differentiation, two additional batteries were selected from previous studies in which non-differentiation had been observed (Liu & Cernat, 2018; Keusch & Yan, 2017). This led to a pool of 59 candidate items for this study.

Building on the empirical methods for judging question and response sensitivity in Feuer and Schober (2015, 2019) and Fail et al. (2021), all items (topics) were rated in an online study for the extent to which most people would be very uncomfortable, somewhat uncomfortable or completely comfortable to be asked each question and to provide each potential response (all categorical responses, and ranges of numeric responses selected based on response distributions from previously published studies [see Fail et al., 2020, p. 6 and endnotes 4 and 5, for more details about this method]). Response options that 50% or more of the raters judged would make most people uncomfortable to answer appear in red. A total of 447 raters recruited by CloudResearch were randomly assigned to rate different subsets of questions and response options, leading to about 150 ratings per topic and response option, in February 2019. These ratings were designed not only to allow for the selection of items to measure disclosure that would indeed be rated as sensitive, but also to verify that items selected to measure rounding and non-differentiation were not unintendedly sensitive.

The Sports battery (which included reverse-worded items, where providing the same answer to contradictory items would be clear evidence of low data quality) was placed later in the questionnaire to allow for potential observation of fatigue effects on non-differentiation. One less sensitive disclosure item (the question about elections) was moved to the final block of questions to promote questionnaire coherence (to after a battery question about government spending on sports).

**Question Text**	**Data Quality Measure**	**Response Options** (*numerical questions were open-ended in actual study; raters were asked to rate sensitivity of ranges of responses based on evidence of prior response distributions)	**Percent of Responses Rated as Very or Somewhat Uncomfortable to Give**	**Study/Survey from Which Question Was Drawn**
On the average day, about how many hours do you personally watch television?	Rounding	0 hours	23.9%	Schober et al. (2015)
		1 hour	17.6%	
		2 hours	15.0%	
		3-5 hours	31.2%	
		6-10 hours	59.0%	
		11-20 hours	65.9%	
During the past 12 months, how many movies have you seen in movie theaters?	Rounding	0 movies	26.1%	Schober et al. (2015)
		1-15 movies	17.5%	
		16-30 movies	40.1%	
		31-55 movies	47.0%	
During the past 12 months, how many movies did you watch in any medium?	Rounding	0 movies	26.2%	Schober et al. (2015)
		1-15 movies	16.9%	
		16-30 movies	31.6%	
		31-50 movies	46.7%	
During the last month, how many times did you eat in restaurants?	Rounding	0 times	24.6%	Schober et al. (2015)
		1-15 times	25.4%	
		16-30 times	55.3%	
		31-50 times	0.0%	

Question Text	Data Quality Measure	Response Options (*numerical questions were open-ended in actual study; raters were asked to rate sensitivity of ranges of responses based on evidence of prior response distributions)	Percent of Responses Rated as Very or Somewhat Uncomfortable to Give	Study/Survey from Which Question Was Drawn
During the last month, how many times did you eat spicy food?	Rounding	0 days	18.7%	Schober et al. (2015)
		1-10 days	21.2%	
		11-20 days	23.0%	
		21-31 days	26.9%	
During the last month, how many times did you shop in a grocery store?	Rounding	0 times	31.0%	Schober et al. (2015)
		1-15 times	12.4%	
		16-30 times	47.2%	
		31-60 times	52.4%	
How many fluid ounces of plain drinking water did you drink yesterday from the tap or water fountain?	Rounding	0 fluid ounces (1)	46.4%	Continuing Survey of Food Intakes by Individuals
		1-16 fluid ounces (2)	33.8%	
		17-32 fluid ounces (3)	24.2%	
		33-48 fluid ounces (4)	29.9%	
		49-64 fluid ounces (5)	27.5%	
		More than 64 fluid ounces (6)	35.9%	
How much do you favor or oppose avoiding "fast food"?	Non-differentiation (Food battery)	Strongly favor	33.3%	Schober et al. (2015)
		Somewhat favor	31.6%	
		Neither favor nor oppose	33.9%	
		Somewhat oppose	42.4%	
		Strongly oppose	40.5%	
How much do you favor or oppose maintaining a healthy diet?	Non-differentiation (Food battery)	Strongly favor	21.8%	Schober et al. (2015)
		Somewhat favor	26.3%	
		Neither favor nor oppose	37.4%	
		Somewhat oppose	61.7%	
		Strongly oppose	67.5%	
How much do you favor or oppose monitoring cholesterol levels closely?	Non-differentiation (Food battery)	Strongly favor	25.8%	Schober et al. (2015)
		Somewhat favor	28.1%	
		Neither favor nor oppose	38.6%	
		Somewhat oppose	54.5%	
		Strongly oppose	59.5%	
How much do you favor or oppose emphasizing the taste of food rather than its nutritional value?	Non-differentiation (Food battery)	Strongly favor	45.3%	Schober et al. (2015)
		Somewhat favor	38.8%	
		Neither favor nor oppose	40.4%	
		Somewhat oppose	46.0%	
		Strongly oppose	46.9%	
How much do you favor or oppose paying close attention to the nutritional information on food packaging?	Non-differentiation (Food battery)	Strongly favor	20.7%	Schober et al. (2015)
		Somewhat favor	20.2%	
		Neither favor nor oppose	34.5%	
		Somewhat oppose	52.2%	
		Strongly oppose	53.0%	
How much do you favor or oppose limiting the amount of red meat in your diet?	Non-differentiation (Food battery)	Strongly favor	32.5%	Schober et al. (2015)
		Somewhat favor	32.5%	
		Neither favor nor oppose	41.7%	
		Somewhat oppose	45.3%	
		Strongly oppose	46.5%	

Question Text	Data Quality Measure	Response Options (*numerical questions were open-ended in actual study; raters were asked to rate sensitivity of ranges of responses based on evidence of prior response distributions)	Percent of Responses Rated as Very or Somewhat Uncomfortable to Give	Study/Survey from Which Question Was Drawn
How much do you favor or oppose balancing one's diet across the key food groups?	Non-differentiation (Food battery)	Strongly favor	20.9%	Schober et al. (2015)
		Somewhat favor	29.8%	
		Neither favor nor oppose	40.5%	
		Somewhat oppose	53.1%	
		Strongly oppose	53.1%	
Please indicate to what extent you agree or disagree with the following statement: It is important to me to have really nice things.	Non-differentiation (Money battery)	Strongly agree	37.5%	Keusch & Yan (2017)
		Somewhat agree	36.1%	
		Neither agree nor disagree	32.1%	
		Somewhat disagree	40.2%	
		Strongly disagree	46.8%	
Please indicate to what extent you agree or disagree with the following statement: I would like to be rich enough to buy anything I want.	Non-differentiation (Money battery)	Strongly agree	24.8%	Keusch & Yan (2017)
		Somewhat agree	24.3%	
		Neither agree nor disagree	27.4%	
		Somewhat disagree	37.7%	
		Strongly disagree	41.8%	
Please indicate to what extent you agree or disagree with the following statement: I'd be happier if I could afford to buy more things.	Non-differentiation (Money battery)	Strongly agree	36.0%	Keusch & Yan (2017)
		Somewhat agree	38.0%	
		Neither agree nor disagree	36.1%	
		Somewhat disagree	43.9%	
		Strongly disagree	43.9%	
Please indicate to what extent you agree or disagree with the following statement: It sometimes bothers me quite a bit that I can't afford to buy all the things I would like.	Non-differentiation (Money battery)	Strongly agree	56.8%	Keusch & Yan (2017)
		Somewhat agree	61.3%	
		Neither agree nor disagree	48.6%	
		Somewhat disagree	53.7%	
		Strongly disagree	52.3%	
Please indicate to what extent you agree or disagree with the following statement: It's really true that money can buy happiness.	Non-differentiation (Money battery)	Strongly agree	57.0%	Keusch & Yan (2017)
		Somewhat agree	58.9%	
		Neither agree nor disagree	49.6%	
		Somewhat disagree	51.4%	
		Strongly disagree	47.3%	
Please indicate to what extent you agree or disagree with the following statement: The things I own give me a great deal of pleasure.	Non-differentiation (Money battery)	Strongly agree	28.2%	Keusch & Yan (2017)
		Somewhat agree	25.7%	
		Neither agree nor disagree	33.0%	
		Somewhat disagree	49.1%	
		Strongly disagree	49.5%	

Question Text	Data Quality Measure	Response Options (*numerical questions were open-ended in actual study; raters were asked to rate sensitivity of ranges of responses based on evidence of prior response distributions)	Percent of Responses Rated as Very or Somewhat Uncomfortable to Give	Study/Survey from Which Question Was Drawn
How often do you pay off the total balance on your credit card(s) each month?	Disclosure	Almost always	34.8%	Survey of Consumer Finances, Survey of Consumer Attitudes
		Sometimes	65.2%	
		Hardly ever	72.4%	
		Not applicable	NA	
How often do you attend religious services?	Disclosure	At least once a week	26.1%	Schober et al. (2015)
		Almost every week	30.5%	
		About once a month	50.8%	
		Seldom	62.6%	
		Never	65.9%	
During the past 12 months, how often have you offered your seat on a bus or in a public place to a stranger who was standing?	Disclosure	Not at all in the past year	54.4%	General Social Survey
		Once in the past year	48.6%	
		Two or three times a year	42.6%	
		Once a month	34.6%	
		Once a week	24.8%	
		More than once a week	23.0%	
		Don't know	42.2%	
During the past 12 months, how often have you done volunteer work for a charity?	Disclosure	Not at all in the past year	68.5%	General Social Survey
		Once in the past year	43.6%	
		Two or three times a year	25.0%	
		Once a month	21.1%	
		Once a week	20.7%	
		More than once a week	59.0%	
		Don't know	NA	
During the past 12 months, how often have you given food or money to a homeless person?	Disclosure	Not at all in the past year	68.9%	General Social Survey
		Once in the past year	48.7%	
		Two or three times a year	34.7%	
		Once a month	33.6%	
		Once a week	37.4%	
		More than once a week	55.1%	
		Don't know	NA	
To what extent do you agree or disagree with the following statement:<br>There is too much sport on TV.	Non-differentiation (Sports battery)	Strongly agree	35.9%	Liu & Cernat (2018)
		Agree	38.5%	
		Somewhat agree	35.8%	
		Neither agree nor disagree	31.8%	
		Somewhat disagree	40.2%	
		Disagree	43.9%	
		Strongly disagree	41.1%	
To what extent do you agree or disagree with the following statement:<br>Sports bring different groups and races inside a country closer together.	Non-differentiation (Sports battery)	Strongly agree	31.9%	Liu & Cernat (2018)
		Agree	31.2%	
		Somewhat agree	36.4%	
		Neither agree nor disagree	46.2%	
		Somewhat disagree	62.6%	
		Disagree	61.2%	
		Strongly disagree	61.0%	

Question Text	Data Quality Measure	Response Options (*numerical questions were open-ended in actual study; raters were asked to rate sensitivity of ranges of responses based on evidence of prior response distributions)	Percent of Responses Rated as Very or Somewhat Uncomfortable to Give	Study/Survey from Which Question Was Drawn
To what extent do you agree or disagree with the following statement: International sports competitions create more tension between countries than good feelings.	Non-differentiation (Sports battery)	Strongly agree	56.8%	Liu & Cernat (2018)
		Agree	57.1%	
		Somewhat agree	51.0%	
		Neither agree nor disagree	48.1%	
		Somewhat disagree	50.0%	
		Disagree	53.4%	
		Strongly disagree	51.4%	
To what extent do you agree or disagree with the following statement: Governments should spend more money on sports.	Non-differentiation (Sports battery)	Strongly agree	53.2%	Liu & Cernat (2018)
		Agree	54.3%	
		Somewhat agree	53.3%	
		Neither agree nor disagree	49.5%	
		Somewhat disagree	49.0%	
		Disagree	47.6%	
		Strongly disagree	47.1%	
What about local elections – do you always vote in those, do you sometimes miss one, do you rarely vote, or do you never vote?	Disclosure	Always vote	23.0%	General Social Survey
		Sometimes miss one	45.8%	
		Rarely vote	71.8%	
		Never vote	73.9%	
About how often did you have sex during the last 12 months?	Disclosure	Not at all	85.0%	Schober et al. (2015); General Social Survey
		Once or twice	83.4%	
		About once a month	75.3%	
		2 or 3 times a month	70.3%	
		Once a week	65.5%	
		2 or 3 times a week	67.6%	
		4 or more times a week	66.0%	
How many sex partners have you had in the last 12 months?	Disclosure	0	59.73%	Schober et al. (2015); General Social Survey
		1	47.30%	
		2	75.32%	
		3-4	85.42%	
		5-6	88.89%	
		7-8	90.92%	
		9-10	91.67%	
Now thinking about the time since your 18th birthday, how many female partners have you had sex with?	Disclosure	0	71.62%	Schober et al. (2015); General Social Survey
		1	67.61%	
		2	73.61%	
		3-5	81.25%	
		6-10	88.65%	
		11-20	88.57%	
		21-100	87.86%	

Question Text	Data Quality Measure	**Response Options** (*numerical questions were open-ended in actual study; raters were asked to rate sensitivity of ranges of responses based on evidence of prior response distributions)	Percent of Responses Rated as Very or Somewhat Uncomfortable to Give	Study/Survey from Which Question Was Drawn
Thinking about the time since your 18th birthday, how many male partners have you had sex with?	Disclosure	0	57.24%	Schober et al. (2015); General Social Survey
		1	61.81%	
		2	72.22%	
		3-5	85.21%	
		6-10	90.14%	
		11-20	91.43%	
		21-100	90.78%	
During the past 12 months, have your sex partners been exclusively male, exclusively female, both male and female, or have you had no partners?	Disclosure	Exclusively male	81.2%	Schober et al. (2015); General Social Survey
		Exclusively female	48.3%	
		Both male & female	89.4%	
		No partners	69.3%	
In the past 30 days, how often have you visited a website for sexually explicit material?	Disclosure	Never	39.5%	General Social Survey
		1-2 times	79.4%	
		3-5 times	87.7%	
		More than 5 times	88.7%	

Supplementary Appendix C

Online debriefing items administered immediately after respondents completed the survey

Question	Response Options
Overall, how satisfied were you with this survey?	1. Not at all satisfied<br>2.<br>3.<br>4.<br>5. Very satisfied
[Live Video and Prerecorded Video]<br><br>Overall, how comfortable were you with the interviewer?	1. Not at all comfortable<br>2.<br>3.<br>4.<br>5. Very comfortable
[Live Video]<br><br>How much did you enjoy interacting with the interviewer?	1. Did not enjoy at all<br>2.<br>3.<br>4.<br>5. Thoroughly enjoyed
[Live Video and Prerecorded Video]<br><br>How personally connected did you feel to the interviewer?	1. Distant<br>2.<br>3.<br>4.<br>5. Close
How often did you feel that you were able to answer the questions honestly?	– Always<br>– Most of the time<br>– Some of the time<br>– Never
Imagine you had been asked the survey questions in person, that is, in a face-to-face interview. Did the survey you just completed feel more private, the same, or less private than being asked the questions face-to-face?	– More private<br>– The same<br>– Less private
Did anyone nearby affect the way you answered the questions?	– No, the people nearby did not affect my answers<br>– Yes, the people nearby affected my answers<br>– No one was around
How sensitive did you feel the survey questions were?	1. Not at all sensitive<br>2.<br>3.<br>4.<br>5. Very sensitive
[Live Video and Prerecorded Video]<br><br>Did you experience any of the following at any point during the interview?<br><br>No audio<br>Distorted or muffled speech<br>Background noise<br>Echo<br>Volume too soft<br>Interrupted speech<br>(i.e., you and the interviewer were speaking at the same time) [VM]<br>No video<br>Frozen or distorted video<br>Trouble seeing what was on the screen clearly<br>Video and audio out of sync<br>Other technical problem (please specify)	For each item:<br>– Yes<br>– No

Question	Response Options
IF YES TO ANY OF THE ABOVE:<br><br>Was the [problem] resolved?	– No<br>– Yes, it resolved itself<br>– Yes, I was able to resolve it myself<br>– Yes, someone helped me resolve it
How often do you participate in live video calls on any device?	– More than once a week<br>– Weekly<br>– Monthly<br>– A few times a year<br>– Once a year<br>– Seldom or never
Were you doing something else during the interview?	– Yes<br>– No
IF YES:<br><br>What else were you doing during the interview?	[open-ended]
If there is anything else you would like to mention about your experience with the interviewer, please record it here.	[open-ended]
What is the highest level of education that you have completed?	– Not a high school graduate<br>– High school graduate (or GED)<br>– Some college<br>– Vocational or associate degree<br>– Bachelor's degree<br>– Graduate degree
What is your annual household income from all sources?	– $25,000 or less<br>– $25,000 to $50,000<br>– $50,000 to $75,000<br>– $75,000 or more<br>– Decline to answer
How do you identify yourself?	– Male<br>– Female<br>– Nonbinary
How do you identify yourself? [check all that apply]	– Black or African American<br>– White<br>– American Indian, Native American, or Alaskan Native<br>– Asian<br>– Native Hawaiian or Other Pacific Islander<br>– Other (specify)
How many people (including yourself) are living or staying at this address?	[open-ended]

Supplementary Appendix D

Interviewer training details

Interviewers were trained for live video interviewing as a group in a single session. They were instructed to follow standardized interviewing procedures (the University of Michigan's General Interviewer Training). They were also instructed about technical aspects of the BlueJeans video platform and on using the Blaise survey software (which they all regularly used) on the same screen. This included training interviewers on fixes that often work for a small set of technical issues that we thought a priori were most likely to occur (e.g., advising respondents to check microphone or camera connections, reconnecting to the video call or advising the respondent to reconnect, or even rebooting either participants' device). We also provided resources for interviewers for obtaining additional technical support and for rescheduling if necessary.

Interviewers were also trained on protocols for following up via email with a respondent who did not show up to a scheduled interview after 5 minutes, indicating that they would keep the video meeting open and be available to conduct the interview for another 10 minutes. They were asked to complete a brief post-interview questionnaire after each video interview to document any technical issues that had occurred from their perspective.

Supplementary Appendix E

Invitations and completions by sample source

Final sample sizes in the three modes for both sample sources. For all sample sources, "Invitations" is the number of initial personal invitations requesting participation that were sent. The percentages reported here should not be confused with any AAPOR response rate. For both CloudResearch and MICHR, this percentage is more appropriately characterized as a participation rate (The American Association for Public Opinion Research 2016) or completion rate (Callegaro & DiSogra, 2008), as we do not know how many people were exposed to the study invitation(s), only how many were issued unique links to the survey instrument.

			Mode			
			Live Video	Prerecorded Video	Web	Total
Sample Source	CloudResearch	Invitations	5500	1120	445	7065
		Completes	76	303	337	716
		Rate	1.42%	27.05%	75.73%	10.13%
	MICHR	Invitations	283	105	97	485
		Completes	203	82	66	351
		Rate	71.73%	78.10%	68.04%	72.37%
Total		Invitations	5783	1225	542	7550
		Completes	279	385	403	1067
		Rate	4.82%	31.43%	74.35%	14.13%

Supplementary Appendix F

Sample Composition by Gender, Age, Race and Education. Sample size appears in parentheses

Gender	Age	Race	Education	2018 CPS Proportion	Live Video Proportion n=276	Prerecorded Video Proportion n=380	Web Proportion n=402
Male	< 65 years	White	HS and less	0.12	0.025 (7)	0.065 (25)	0.08 (34)
			More than HS	0.18	0.18 (49)	0.13 (48)	0.12 (47)
		Non-white	HS and less	0.04	0.02 (5)	0.02 (8)	0.02 (10)
			More than HS	0.05	0.04 (12)	0.04 (17)	0.04 (16)
	>=65 years	White	HS and less	0.03	0.01 (2)	0.05 (18)	0.04 (18)
			More than HS	0.05	0.07 (21)	0.07 (27)	0.08 (31)
		Non-white	HS and less	0.01	0.00 (0)	0.01 (4)	0.01 (3)
			More than HS	0.01	0.01 (4)	0.02 (7)	0.01 (5)
Female	< 65 years	White	HS and less	0.10	0.065 (18)	0.06 (24)	0.06 (26)
			More than HS	0.20	0.315 (87)	0.24 (91)	0.23 (93)
		Non-white	HS and less	0.04	0.01 (4)	0.02 (7)	0.02 (8)
			More than HS	0.06	0.08 (22)	0.09 (34)	0.05 (19)
	>=65 years	White	HS and less	0.04	0.03 (9)	0.07 (26)	0.10 (39)
			More than HS	0.05	0.10 (28)	0.08 (32)	0.09 (38)
		Non-white	HS and less	0.01	0.01 (2)	0.015 (6)	0.01 (6)
			More than HS	0.01	0.02 (6)	0.02 (6)	0.02 (9)

Note: Differences in numbers of cases from totals reported elsewhere are due to incomplete demographic data (9 total cases).

Supplementary Appendix G

Recruitment Procedure, Incentives and Scheduling

CloudResearch recruits participants from multiple online opt-in panels (Prime Panels). In the current study, CloudResearch invited panelists who had confirmed being 18 years of age or older to complete the survey, offering them $5 or the type and amount of compensation (reward points, gift cards) to which they had previously agreed to with their panel. Our difficulty recruiting sample members to participate in live video interviews using an ABS approach raised the possibility that the participation rate in this mode might also be lower in live video than the other modes in opt-in sample sources. Thus we asked CloudResearch to randomly assign panelists (prior to their answering any survey questions) to live video interviews at a higher rate than the other modes, initially inviting 60% to live video, 20% to prerecorded video, 20% to the web survey. The number of invitations to participate in each mode increased until we reached our target or, in the case of live video, were no longer able to produce completed cases. Sample members assigned to the live video mode were promised an additional $15 (in the form of an Amazon gift code) for completing the survey, to encourage participation despite 1) the extra task of scheduling an interview for a later time (when the interviewer to whom they had been assigned would be available) and 2) participating using a mode with which they might be unfamiliar and which involved greater social contact than the textual web surveys to which they typically respond. Once data collection in the two self-administered modes was completed, all remaining potential respondents were assigned to live video.

Our target of 280 participants in the prerecorded video and web survey modes was reached quickly, but after 76 participants had completed live video interviews it became apparent that the pool of panelists willing to schedule interviews in this mode had been exhausted. Recruitment for live video was then redirected to the MICHR pool whose members were offered a $20 Amazon gift code to complete a live video interview. Once the target number of live video interviews had been conducted, we recruited additional MICHR participants, offering them the same incentive and randomly assigning them to one of the two self-administered modes; this was done to ensure that mode would not be confounded with sample source and that both sources would be represented in all modes.

Changing our recruitment strategy over the course of data collection led to a corresponding change in how live video interviews were scheduled. Participants from CloudResearch were required to schedule interviews only at times when their randomly assigned interviewer was available (even though this would likely reduce or delay participation.) In our implementation, schedule slots were particularly restricted because of our intention to randomly assign respondents to interviewers during the hours they were available so as to allow measurement of potential interviewer effects (reported elsewhere) even though this might delay or reduce participation.

Supplementary Appendix H

Terms Used in Final Models

Rounding

Overall

Rounding: Mode + Age + VideoExperience + SampleSource

RoundingBinary: Mode + Age + VideoExperience + Sample Source + Mode*Age

Items

TelevisionHours: Mode + Age + Education + SampleSource

MovieTheaterYear: Mode + Age + Gender + Mode*Device

MoviesYear: Mode + Age + SampleSource + Mode*Age

RestaurantsMonth: Mode + Age + SampleSource

SpicyFood: Mode + Age + Race + Gender + SampleSource + Device

GroceryStore: Mode + Age + SampleSource + Device

DrinkingWater ~ Mode + Age + VideoExperience + SampleSource

Non-differentiation

Overall

Straightlining: Mode + Age + Gender + VideoExperience + SampleSource + Mode*Age

Individual Batteries

Food Battery: Mode + Age + Education + Gender + SampleSource + Device

Money Battery: Mode + Age + Gender + SampleSource + Mode*Age

Sports Battery: Mode + Age + VideoExperience + SampleSource

Food Battery Questions

FastFood: Mode + Age + VideoExperience + SampleSource + Device + Mode*Age

HealthyDiet: Mode + Age + Race + VideoExperience + SampleSource + Device

Cholesterol: Mode + Age + Education + Race + SampleSource

NutritionalValue: Mode + Age + Gender + SampleSource + Mode*Age

FoodPackaging: Mode + Age + Education + VideoExperience + SampleSource + Mode*Age

MeatDiet: Mode + Age + Gender + VideoExperience + SampleSource + Mode*Age

FoodGroups: Mode + Age + Education + VideoExperience + SampleSource

Money Battery Statements

NiceThings: Mode + Age + Race + VideoExperience + SampleSource

BuyAnything: Mode + Age + Race + Gender + SampleSource

BuyMoreThings: Mode + Age + Race + Gender + SampleSource

Bother: Mode + Age + Education + Race + SampleSource

Happiness: Mode + Age + VideoExperience + SampleSource

Pleasure: Mode + Age + Race + Gender + VideoExperience + SampleSource

Sports Battery Statements

TooMuch: Mode + Age + SampleSource + Device

Race: Mode + Age + Gender + VideoExperience + SampleSource + Mode*Age

International: Mode + Age + Race + Gender + VideoExperience + SampleSource

Government: Mode + Age + Gender + VideoExperience + SampleSource

Disclosure

Overall

SensitivityProportion: Mode + Age + Education + Gender + VideoExperience + SampleSource + Device + Mode*Age

SensitivityScore: Mode + Age + Education + Gender + SampleSource + VideoExperience

Items

CreditCardBalance: Mode + Age + Race + VideoExperience + SampleSource

ReligiousAttendance: Mode + Age + Race + Gender + VideoExperience + SampleSource

BusSeat: Mode + Age + VideoExperience + SampleSource + Device

VolunteerWork: Mode + Age + Education + Gender + VideoExperience + SampleSource

HelpHomeless: Mode + Age + VideoExperience + SampleSource + Device

LocalElections: Mode + Age + Education + Race + VideoExperience + SampleSource

SexPartnersYear: Mode + Age + VideoExperience + SampleSource

FemaleSexPartners: Mode + Age + Education + Gender + SampleSource

MaleSexPartners: Mode + Age + Race + Gender + VideoExperience + SampleSource + Mode*Age

SexFrequency: Mode + Age + Education + Gender + VideoExperience + SampleSource + Device

SexPartnerGender: Mode + Age + VideoExperience + SampleSource + Device

PornFrequency: Mode + Age + Gender + SampleSource

methods, data, analyses | 2022, pp. 1-23

Supplementary Appendix I

Numerical Estimates by Mode

Numerical estimates by mode. Comparisons were calculated by fitting GEE models adjusting for age, sample source, and interviewer cluster, excluding responses of zero; for the pairwise comparisons with live video, live video was the reference category, and for the comparison between web survey and prerecorded video the reference category was recorded video.

		Live Video (n = 276)	Web Survey (n = 403)	Prerecorded video (n = 383)	Live Video vs. Web Survey	Live Video vs. Prerecorded Video	Prerecorded Video vs. Web Survey
Item		Estimates			p-value		
TelevisionHours	mean	6.573 (0.533)	7.613 (0.529)	7.398 (0.499)	**<0.001**	**<0.001**	0.668
MovieTheaterYear	mean	6.645 (0.625)	5.197 (0.463)	5.726 (0.718)	**0.026**	0.156	0.248
MoviesYear	mean	38.184 (1.115)	30.610 (4.410)	7.708 (4.264)	0.120	**<0.001**	**<0.001**
RestaurantsMonth	mean	6.269 (0.629)	3.541 (0.714)	3.614 (0.755)	**<0.001**	**<0.001**	0.496
SpicyFood	mean	5.658 (0.271)	5.652 (1.114)	4.404 (0.899)	0.995	0.132	0.226
GroceryStore	mean	7.505 (0.559)	9.908 (1.944)	7.822 (1.238)	0.203	0.334	0.207
DrinkingWater	mean	27.132 (1.699)	26.395 (1.461)	25.947 (3.195)	0.825	0.727	0.840

Supplementary Appendix J

Percent "Strongly Favor" or "Strongly Agree" by mode; differences presumably due at least in part to mode differences in non-differentiation

Estimates (e.g., percent of respondents choosing "strongly favor" in judging battery statements) by mode. For the pairwise comparisons with Live Video, Live Video was the reference category, and for the comparison between Web Survey and Prerecorded Video the reference category was prerecorded video.

		Live Video (n = 273)	Web Survey (n = 402)	Prerecorded Video (n = 383)	Live Video vs. Web Survey	Live Video vs. Prerecorded Video	Prerecorded Video vs. Web Survey
		Estimates			p-value		
**FoodBattery**							
FoodBattery_FastFood	Strongly favor	9.1% (24.2%*)	5.7% (21.7%*)	11.7% (20.1%*)	0.666	0.004	0.002
FoodBattery_HealthyDiet	Strongly favor	54.3% (20.4%)	39.8% (21.5%)	45.3% (26.8%)	0.001	0.055	0.118
FoodBattery_Cholesterol	Strongly favor	43.4% (19.6%)	37.0% (19.8%)	38.1% (28.2%)	0.247	0.332	0.788
FoodBattery_Nutritional Value	Strongly favor	31.2% (11.3%)	17.4% (16.8%)	22.6% (28.7%*)	0.135	0.810	0.048
FoodBattery_Food Packaging	Strongly favor	36.0% (17.0%)	27.9% (13.6%)	34.2% (18.5%)	0.659	0.089	0.018
FoodBattery_MeatDiet	Strongly favor	10.3% (20.4%*)	13.1% (19.1%*)	13.2% (24.9%*)	0.205	0.030	0.543
FoodBattery_FoodGroups	Strongly favor	42.6% (11.5%)	28.5% (13.9%)	31.2% (20.2%)	0.001	0.009	0.421
**MoneyBattery**							
MoneyBattery_NiceThings	Strongly agree	18.3% (22.3%*)	21.6% (25.4%*)	24.8% (30.6%*)	0.520	0.188	0.401
MoneyBattery_BuyAnything	Strongly agree	38.9% (19.2%)	56.8% (23.2%)	58.9% (21.8%)	0.000	0.000	0.638
MoneyBattery_BuyMoreThings	Strongly agree	38.4% (24.2%)	40.3% (24.8%)	44.4% (27.3%)	0.745	0.308	0.354
MoneyBattery_Bother	Strongly agree	17.5% (28.2%*)	27.6% (26.1%)	25.2% (32.8%*)	0.042	0.131	0.545
MoneyBattery_Happiness	Strongly agree	2.3% (30.4%*)	5.5% (29.4%*)	6.4% (50.0%*)	0.059	0.028	0.541
MoneyBattery_Pleasure	Strongly agree	34.3% (27.9%)	29.0% (27.3%)	33.8% (32.3%)	0.243	0.908	0.191
**SportsBattery**							
SportsBattery_TooMuch	Strongly agree	13.4% (19.0%*)	12.7% (16.7%*)	18.2% (23.6%*)	0.807	0.184	0.061
SportsBattery_Race	Strongly agree	39.6% (15.5%)	18.5% (17.4%)	27.1% (24.0%)	0.001	0.313	0.001
SportsBattery_International	Strongly agree	6.2% (59.0%*)	4.0% (72.3%*)	7.0% (75.2%*)	0.333	0.790	0.156
SportsBattery_Government	Strongly agree	5.8% (46.1%*)	2.7% (46.3%*)	2.3% (51.3%*)	0.116	0.085	0.700

methods, data, analyses | 2022, pp. 1-23

Supplementary Appendix K

Mean sensitivity of the selected responses for each item, where sensitivity is measured by the percent of online raters judging each response as very or somewhat uncomfortable for most people to give

Comparisons were calculated by fitting GEE models (in Stata 16.0) adjusting for age, sample source, education, gender, video experience, device type, and interviewer cluster. For the pairwise comparisons with live video, live video was the reference category, and for the comparison between web survey and prerecorded video the reference category was recorded video.

		Live Video (n = 279)	Web Survey (n = 403)	Prerecorded Video (n = 385)	Live Video vs. Web Survey	Live Video vs. Prerecorded Video	Prerecorded Video vs. Web Survey
						p-value	
CreditCardBalance	Mean response sensitivity	0.559 (0.018)	0.576 (0.017)	0.565 (0.016)	0.050	**0.017**	0.815
ReligiousAttendance	Mean response sensitivity	0.561 (0.007)	0.578 (0.006)	0.565 (0.009)	**0.038**	**0.030**	0.780
BusSeat	Mean response sensitivity	0.438 (0.005)	0.442 (0.004)	0.450 (0.004)	0.586	0.079	0.125
VolunteerWork	Mean response sensitivity	0.472 (0.016)	0.528 (0.012)	0.548 (0.020)	**0.003**	**<0.001**	0.217
HelpHomeless	Mean response sensitivity	0.474 (0.012)	0.513 (0.010)	0.539 (0.019)	**0.029**	**0.001**	**0.029**
LocalElections	Mean response sensitivity	0.406 (0.018)	0.432 (0.020)	0.430 (0.025)	0.718	0.941	0.558
SexPartnersYear	Mean response sensitivity	0.592 (0.016)	0.583 (0.016)	0.594 (0.016)	0.217	0.782	0.186
FemaleSexPartners	Mean response sensitivity	0.817 (0.007)	0.817 (0.007)	0.811 (0.007)	0.911	0.185	0.130
MaleSexPartners	Mean response sensitivity	0.519 (0.007)	0.598 (0.009)	0.598 (0.012)	0.176	0.080	0.865
SexFrequency	Mean response sensitivity	0.764 (0.005)	0.760 (0.006)	0.771 (0.008)	0.589	0.405	**0.036**
SexPartnerGender	Mean response sensitivity	0.535 (0.011)	0.530 (0.009)	0.537 (0.010)	0.139	0.376	0.546
PornFrequency	Mean response sensitivity	0.615 (0.013)	0.655 (0.015)	0.659 (0.016)	**0.004**	**0.001**	0.783

Supplementary Appendix L

Prevalence Estimates for Sensitive Behaviors by Mode

Comparisons were calculated by fitting GEE models (in Stata 16.0) adjusting for age, sample source, education, gender, video experience, device type, and interviewer cluster. For the pairwise comparisons with live video, live video was the reference category, and for the comparison between web survey and prerecorded video the reference category was prerecorded video.

		Live Video (n = 273)	Web Survey (n = 402)	Prerecorded Video (n = 381)	Live Video vs. Web Survey	Live Video vs. Prerecorded Video	Prerecorded Video vs. Web Survey
						p-value	
**Percentage of respondents reporting most sensitive response**		55.1% (1.5%)	55.7% (1.1%)	56.2% (1.6%)	0.053	**0.004**	0.148
Credit Card Balance	Hardly ever	29.6% (26.3%)	30.9% (23.4%)	27.9% (27.2%)	0.761	0.659	0.413
Religious Attendance	Never	18.6% (25.7%*)	23.7% (29.3%*)	26.9% (31.6%*)	0.072	**0.007**	0.228
BusSeat	Not at all in the past year	36.8% (20.4%)	33.5% (15.8%)	43.4% (28.1%)	0.566	0.281	**0.023**
VolunteerWork	Not at all in the past year	41.1% (17.1%)	58.8% (15.4%)	66.2% (26.3%)	**0.006**	**<0.001**	**0.032**
HelpHomeless	Not at all in the past year	30.2% (16.3%)	40.0% (13.2%)	46.1% (26.9%)	0.089	**0.016**	0.157
LocalElections	Never vote	7.7% (39.4%*)	12.3% (37.3%*)	11.9% (41.8%*)	0.087	0.072	0.859
SexFrequency	Not at all	31.1% (16.7%)	28.4% (19.2%)	30.4% (27.6%)	0.564	0.869	0.547
SexPartnerGender	Both male and female	0.6% (101.2%*)	0.5% (86.3%*)	0.4% (93.5%*)	0.181	1.000	0.799
PornFrequency	More than five times in last 30 days	14.9% (13.2%)	25.6% (16.9%)	24.7% (20.5%)	**0.006**	**0.001**	0.812
SexPartnersYear	7 partners or more	0.3% (63.5%*)	0.5% (79.8%*)	0.5% (114.0%*)	0.474	0.464	0.972
FemaleSexPartners	21 or more	27.1% (27.6%*)	16.5% (21.1%*)	12.2% (25.9%*)	0.056	**0.008**	0.305
MaleSexPartners	21 or more	0.6% (69.2%*)	0.7% (58.0%*)	0.6% (67.0%*)	0.434	0.247	0.699

Supplementary Appendix M

Technical Problems

Percentage of reported technical problems in Live Video interviewees by respondents (n=279) or interviewers (n=9) that occurred at least once at any point during the interview, from online debriefing survey.

Technical problem	Proportion of interviews in which respondent reported problem	Proportion of interviews in which interviewer reported problem	% problems resolved as reported by respondent	% problems resolved without intervention as reported by respondent
No audio	14.0% (n=39)	13.7% (n=38)	97.4% (n=38)	44.7% (n=17)
Distorted or muffled speech	14.0% (n=39)	11.5% (n=32)	94.9% (n=37)	70.3% (n=26)
Background Noise	6.5% (n=18)	4.7% (n=13)	66.7% (n = 12)	100.0% (n = 12)
Echo	3.6% (n=10)	1.8% (n=5)	80.0% (n = 8)	75.0% (n=6)
Volume too soft	2.5% (n=7)	5.7% (n=16)	71.4% (n=5)	60.0% (n=3)
Interrupted speech (interviewer and respondent were speaking at the same time)	18.3% (n=51)	9.0% (n=25)	94.1% (n=48)	68.8% (n=33)
No video	9.0% (n=25)	16.8% (n=47)	92.0% (n=23)	30.4% (n=7)
Frozen or distorted video	11.5% (n=32)	10.8% (n=30)	90.6% (n=29)	72.4% (n=21)
Trouble seeing what was on the screen clearly	17.6% (n=49)	3.9% (n=11)	85.7% (n=42)	76.2% (n=32)
Video and audio out of sync	17.9% (n=50)	14.0% (n=39)	72.0% (n=36)	63.9% (n=23)

References

American Association for Public Opinion Research. (2016). Standard Definitions: Final Dispositions of Case Codes and Outcome Rates for Surveys. 9th edition.

Callegaro, M., & DiSogra, C. (2008). Computing response metrics for online panels. *Public opinion quarterly*, 72(5), 1008-1032. https://doi.org/10.1093/poq/nfn065

Fail, S., Schober, M. F., & Conrad, F. G. (2021). The time it takes to reveal embarrassing information in a mobile phone survey. *International journal of social research methodology*, 24(2), 249-264. https://doi.org/10.1080/13645579.2020.1824629

Feuer, S., & Schober, M.F. (2015, May 21-24). Self-view can increase disclosure in Skype survey interviews. 27th Annual Convention of the Association for Psychological Science, New York, NY, United States.

Feuer, S., & Schober, M.F. (2019, July 15-19). Gaze patterns and the self-view window in Skype survey interviews. 8th Conferenceof the European Survey Research Association, Zagreb, Croatia.

Lind, L. H., Schober, M. F., Conrad, F. G., & Reichert, H. (2013). Why do survey respondents disclose more when computers ask the questions? *Public Opinion Quarterly*, *77*(4), 888–935. https://doi.org/10.1093/poq/nft038

Liu, M., & Cernat, A. (2018). Item-by-item versus matrix questions: A web survey experiment. *Social Science Computer Review*, 36(6), 690-706. https://doi.org/10.1177/0894439316674459

Keusch, F., & Yan, T. (2017). Web versus mobile web: An experimental study of device effects and self-selection effects. *Social Science Computer Review*, 35(6), 751-769. https://doi.org/10.1177/0894439316675566

Schober, M. F., Conrad, F. G., Antoun, C., Ehlen, P., Fail, S., Hupp, A. L., Johnston, M., Vickers, L., Yan, H.Y., & Zhang, C. (2015). Precision and disclosure in text and voice interviews on smartphones. *PloS one*, 10(6), e0128337. https://doi.org/10.1371/journal.pone.0128337

Date of online first publication: 2022-10-25
